# Experimental and Theoretical Study of Ni^II^‐ and Pd^II^‐Promoted Double Geminal C(sp^3^)−H Bond Activation Providing Facile Access to NHC Pincer Complexes: Isolated Intermediates and Mechanism

**DOI:** 10.1002/chem.202200507

**Published:** 2022-06-13

**Authors:** Fengkai He, Christophe Gourlaouen, Huan Pang, Pierre Braunstein

**Affiliations:** ^1^ School of Chemistry and Chemical Engineering Yangzhou University Yangzhou 225009 Jiangsu P. R. China) E-mail: s; ^2^ Laboratoire de Chimie de Coordination Institut de Chimie (UMR 7177 CNRS) Université de Strasbourg 4 rue Blaise Pascal 67081 Strasbourg France; ^3^ Laboratoire de Chimie Quantique Institut de Chimie (UMR 7177 CNRS) Université de Strasbourg 4 rue Blaise Pascal 67081 Strasbourg France

**Keywords:** alkane activation, C−H bond activation, density functional calculations, N-heterocyclic carbenes, pincer ligands

## Abstract

We report the first examples of metal‐promoted double geminal activation of C(sp^3^)−H bonds of the N−CH_2_−N moiety in an imidazole‐type heterocycle, leading to nickel and palladium N‐heterocyclic carbene complexes under mild conditions. Reaction of the new electron‐rich diphosphine 1,3‐bis((di‐*tert*‐butylphosphaneyl)methyl)‐2,3‐dihydro‐1*H*‐benzo[*d*]imidazole (**1**) with [PdCl_2_(cod)] occurred in a stepwise fashion, first by single C−H bond activation yielding the alkyl pincer complex [PdCl(PC${}_{\mathrm{sp}{}^{3}}$ ^H^P)] (**3**) with two *trans* phosphane donors and a covalent Pd−C${}_{\mathrm{sp}{}^{3}}$ bond. Activation of the C−H bond of the resulting α‐methine C${}_{\mathrm{sp}{}^{3}}$ H−M group occurred subsequently when **3** was treated with HCl to yield the NHC pincer complex [PdCl(PC^NHC^P)]Cl (**2**). Treatment of **1** with [NiBr_2_(dme)] also afforded a NHC pincer complex, [NiBr(PC^NHC^P)]Br (**6**), but the reactions leading to the double geminal C−H bond activation of the N−CH_2_−N group were too fast to allow identification or isolation of an intermediate analogous to **3**. The determination of six crystal structures, the isolation of reaction intermediates and DFT calculations provided the basis for suggesting the mechanism of the stepwise transformation of a N−CH_2_−N moiety in the N−C^NHC^−N unit of NHC pincer complexes and explain the key differences observed between the Pd and Ni chemistries.

## Graphical abstract

**A** ***gem*** **of a reaction**: The first examples of stepwise (Pd) or direct (Ni) metal‐promoted double geminal activation of C${}_{\mathrm{sp}{}^{3}}$ −H bonds leading to imidazole‐type N‐heterocyclic carbene metal pincer complexes under mild conditions are presented.

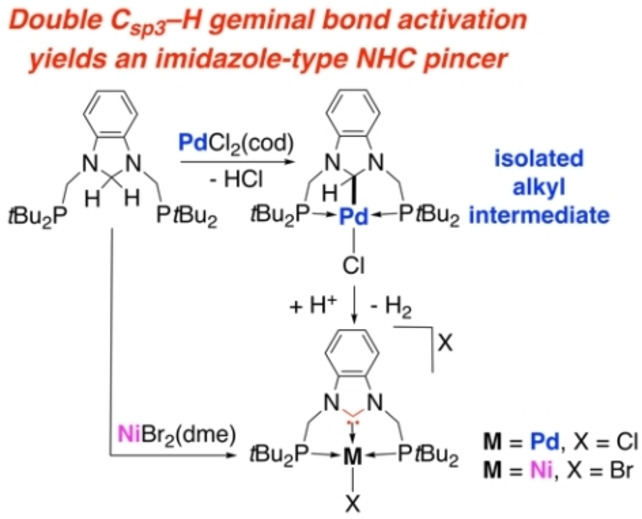

## Introduction

Pincer‐type ligands, defined as tridentate ligands that bind to a metal centre in a meridional fashion, have become ubiquitous in various branches of chemistry, largely because of the easy tunability of their stereoelectronic properties.[^1^, ^2^, ^3^, ^4^, ^5^] These ligands allow, inter alia, the stabilization of metal complexes in different oxidation states, as in the case of Fe,^[6]^ Co^[7]^ and Ni,[^7^, ^8^] and successive electron transfer processes.^[9]^ The relevance of pincer ligands is particularly notable in organometallic chemistry where the search for new physical properties, reactivity patterns and improved catalytic properties remains topical.[^2^, ^3^, ^4^, ^10^, ^11^, ^12^, ^13^, ^14^, ^15^, ^16^, ^17^, ^18^, ^19^, ^20^] In view of the very broad impact of N‐heterocyclic carbene (NHC) chemistry,[^7^, ^8^, ^21^, ^22^, ^23^, ^24^, ^25^, ^26^, ^27^, ^28^, ^29^, ^30^, ^31^, ^32^, ^33^, ^34^, ^35^, ^36^, ^37^, ^38^, ^39^, ^40^, ^41^] it is not surprising that including NHC donor(s) in pincer‐type architectures has become topical in ligand design. The chemical diversity resulting from this combination rapidly made a considerable impact. The relatively easy functionalization of pincer ligands and of NHC donors provides access to a great diversity of ligands and facilitates the fine‐tuning of the properties of their metal complexes.[^2^, ^3^, ^4^, ^5^, ^7^, ^8^, ^13^, ^15^, ^16^, ^19^, ^41^, ^42^, ^43^, ^44^, ^45^, ^46^, ^47^, ^48^, ^49^] The steric properties of NHCs can typically be modified by the choice of the N‐substituents (or wingtip substituents) or the size of the heterocycle, whereas electronic properties are influenced by the nature and number of endocyclic heteroatoms, the site of attachment of the metal on the ring, the electronic saturation or unsaturation of the heterocycle, and a possible annellation resulting from fusing an additional ring on the basic framework.^[50]^

Most synthetic methods leading to NHC complexes or to NHC‐containing pincer complexes involve i) deprotonation of azolium salts to give a free or a coordinated NHC ligand (when a metal‐bound ligand acts as an internal base),[^7^, ^8^, ^41^, ^51^, ^52^, ^53^] ii) transmetallation from silver, copper or mercury reagents,[^7^, ^8^, ^23^, ^54^, ^55^, ^56^, ^57^] both approaches occur without modification of the metal oxidation state,[^3^, ^7^, ^8^, ^23^, ^41^] or iii) oxidative addition of an azolium C${}_{\mathrm{sp}{}^{2}}$ (2)−H bond across a zerovalent metal precursor, which was introduced about 20 years ago.[^58^, ^59^, ^60^, ^61^, ^62^, ^63^]

**Scheme 1:**
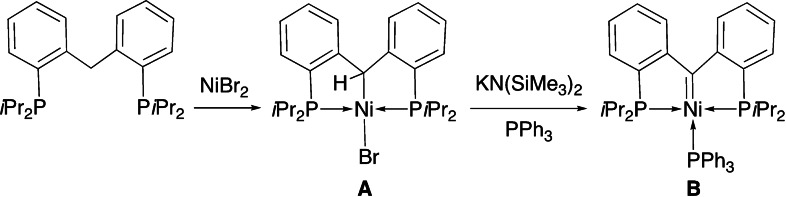
Deprotonation of C${}_{\mathrm{sp}{}^{3}}$ −H in the alkyl complex **A** affords the (bis(2‐(diisopropylphosphaneyl)phenyl)methylene) Ni^II^ pincer complex **B**.^[79]^

The activation and functionalization of C${}_{\mathrm{sp}{}^{3}}$ −H bonds remain targets of considerable interest but are often challenging because of the low reactivity associated to the non‐acidic character of these bonds.[^64^, ^65^, ^66^, ^67^] Among the diverse C−H activation methods available,[^64^, ^68^] those leading to carbene‐transition metal complexes,^[69]^ or promoted by NHC‐transition metal complexes have been recently reviewed.[^5^, ^70^] Palladium complexes are most competent for achieving the activation/functionalization of non‐activated C${}_{\mathrm{sp}{}^{3}}$ −H bonds and because of steric hindrance during the C−H metalation step, methylene C${}_{\mathrm{sp}{}^{3}}$ −H bonds are generally more difficult to functionalize than primary C${}_{\mathrm{sp}{}^{3}}$ −H bonds.^[67]^ The first report of the double C${}_{\mathrm{sp}{}^{3}}$ −H bond activation of a methylene group described the reaction of an alkyl diphosphine with IrCl_3_ to give pincer carbene complexes.^[71]^ Other PCP pincer complexes in which the central donor has carbene character have been successfully prepared, often in moderate yield, by double C−H bond activation with Fe^0^,^[72]^ Ru^II^,[^73^, ^74^, ^75^] Os^IV^,^[74]^ Rh^I^,[^76^, ^77^] Ir^I^,[^76^, ^78^] Ni^II^,^[79]^ and Pd^II^ precursor complexes.[^80^, ^81^] In the case of the proligand bis(2‐(diisopropylphosphaneyl)phenyl)methane, Ni^II^ alkyl complexes of type (**A**) are formed first, which have similarities to complexes with a C${}_{\mathrm{sp}{}^{3}}$ −Pd bond discussed below, and subsequent reaction in THF with a strong base in the presence of PPh_3_ afforded the PC^carbene^P pincer complex (**B**; Scheme 1).^[79]^

Considering the large number of readily available neutral heterocycles containing a N−CH_2_−N moiety, the direct, double geminal C${}_{\mathrm{sp}{}^{3}}$ −H bond activation under mild conditions of this methylene group represents an attractive and concise access of general interest to the carbene functionality. This approach can lead to PC^carbene^P pincer complexes and generally requires electron rich phosphorus donors and suitable metals, with the first C−H activation step occurring by oxidative addition or electrophilic activation and the subsequent activation of the α‐methine group by α‐hydride elimination, hydride or proton abstraction.[^5^, ^19^, ^78^, ^82^] It has been shown that PC${}_{\mathrm{sp}{}^{2}}$ P pincer complexes represent interesting platforms for cooperative bond activation reactions,[^11^, ^47^] and redox‐induced reactivity.[^6^, ^9^, ^83^] Double geminal C−H bond activation at a methylene carbon can have a broader impact, as shown with, for example, the formation of OCO pincer complexes from methylenediphenols and Ir^I^ reagents.^[84]^

Examples of double geminal C${}_{\mathrm{sp}{}^{3}}$ −H bond activation of the N−CH_2_−N moiety in functionalized heterocycles yielding NHC‐metal complexes are still very limited, as, for example, with dihydroperimidine and hexahydropyrimidine derivatives giving Ru^II^, Os^II^, Rh^I^, Ir^III^, Ni^II^ or Pd^II^ P−C^NHC^−P pincer complexes.[^76^, ^85^, ^86^, ^87^, ^88^] To the best of our knowledge, the metal‐promoted double geminal C${}_{\mathrm{sp}{}^{3}}$ −H activation of a methylene group in an imidazole‐type heterocycle, leading stepwise or directly to the currently most popular NHC ligands, has not been reported, despite its conceptual simplicity and appeal, and we decided to explore this possibility in nickel and palladium chemistry. Whereas the relevant C−H bond activation reactions leading to the desired NHC complexes were very fast with Ni^II^, intermediates could be isolated and characterized in the case of Pd^II^, allowing a combined experimental and theoretical investigation of the reaction mechanism which established the stepwise sequence of reactions involved in such a double C${}_{\mathrm{sp}{}^{3}}$ −H bond activation and the relative stability of the intermediates leading to NHC complexes.

## Results and Discussion

**Scheme 2:**
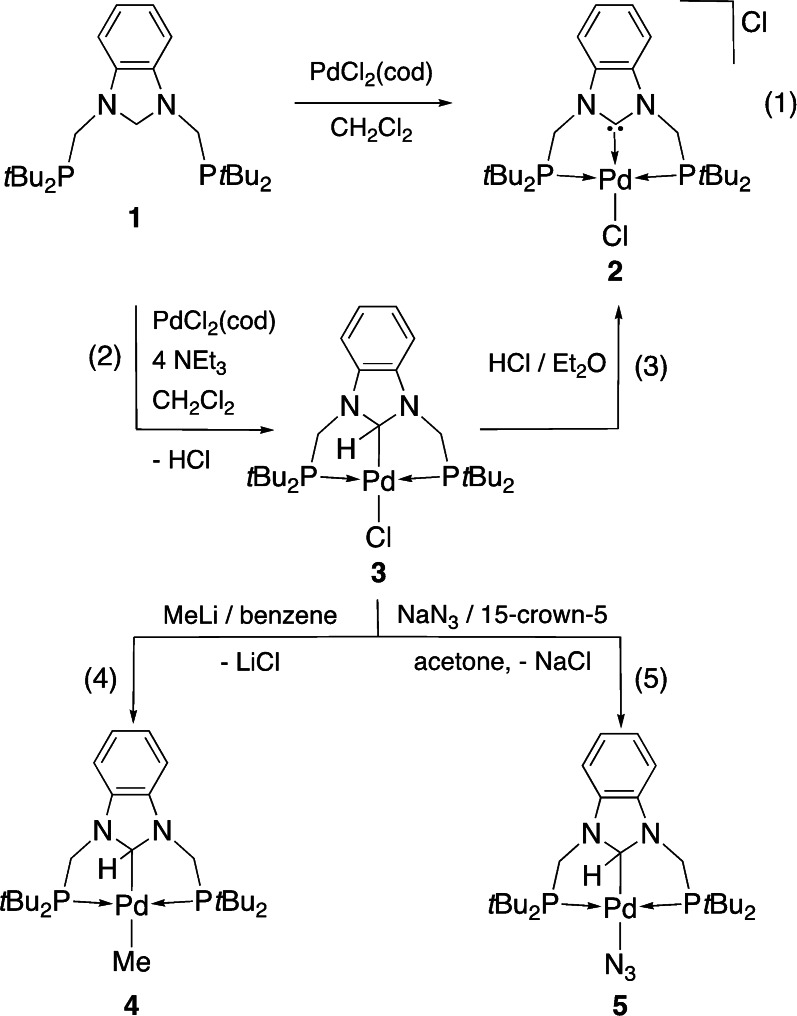
Stepwise double germinal C${}_{\mathrm{sp}{}^{3}}$ −H activation reactions of **1** affording the NHC pincer complex **2** and reactivity of the alkyl pincer intermediate **3**.

We first set out to prepare an electron‐rich diphosphine as potential precursor to a PC^NHC^P pincer system, and we selected 1,3‐bis((di‐*tert*‐butylphosphaneyl)methyl)‐2,3‐dihydro‐1*H*‐benzo[*d*]imidazole (**1**; Scheme 2). The synthesis and molecular structure of this new ligand are detailed in the Supporting Information (Table S1, Figure S17). Using conventional methods, the corresponding benzo[*d*]imidazolium salt and derived free and coordinated (M=Mo, Ru, Ni, Rh, Ir) carbene derivatives have been obtained recently,[^89^, ^90^, ^91^] likewise the less electron‐rich, 1,3‐bis((diphenylphosphaneyl)methyl)‐2,3‐dihydro‐1*H*‐benzo[*d*]imidazole, its corresponding azolium salt and some palladium and rhodium PC^NHC^P pincer complexes.^[92]^

**Figure 1:**
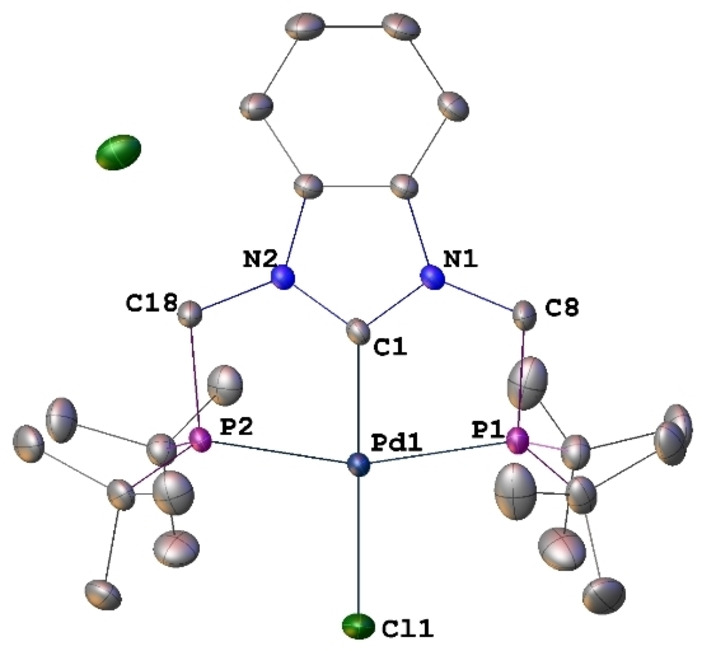
Structure of [PdCl(PC^NHC^P)]Cl (**2**) in **2⋅**CH_2_Cl_2_ with H atoms omitted for clarity. Thermal ellipsoids are shown at 50 % probability. Selected bond lengths [Å] and angles [°]: Pd1−C1 1.940(2), Pd1−P1 2.3363(6), Pd1−P2 2.3413(6), Pd1−Cl1 2.3511(6), C1−N1 1.352(3), C1−N2 1.352(3), C8‐−P1 1.864(2), C18−P2 1.865(2); N1−C1−N2 107.78(19), C1−Pd1−P1 81.25(6), C1−Pd1−P2 81.54(6), C1−Pd1−Cl1 176.85(7), N1−C1−Pd1 126.36(16), N2−C1−Pd1 125.80(15), P1−Pd1−Cl1 98.78(2), P2−Pd1−Cl1 98.65(2), P1−Pd1−P2 162.22(2).

When **1** was treated with [PdCl_2_(cod)] in CH_2_Cl_2_ at room temperature overnight, a white solid **2** was obtained in 70 % yield. Its ^1^H NMR spectrum contained no signal corresponding to the NCH_2_N protons of **1** and ^31^P NMR spectroscopy confirmed P‐coordination to Pd. An X‐ray diffraction analysis established the formulation of **2** as a NHC pincer complex, [PdCl(PC^NHC^P)]Cl, resulting from the double geminal C−H bond activation of the N−CH_2_−N unit of **1** [Eq. (1) in Scheme 2, Figure 1]. The metrical data within the square planar complex **2** are as expected, with a Pd−C^NHC^ bond length of 1.940(2) Å and a C1−Pd1−Cl1 angle of 176.85(7)°.

**Figure 2:**
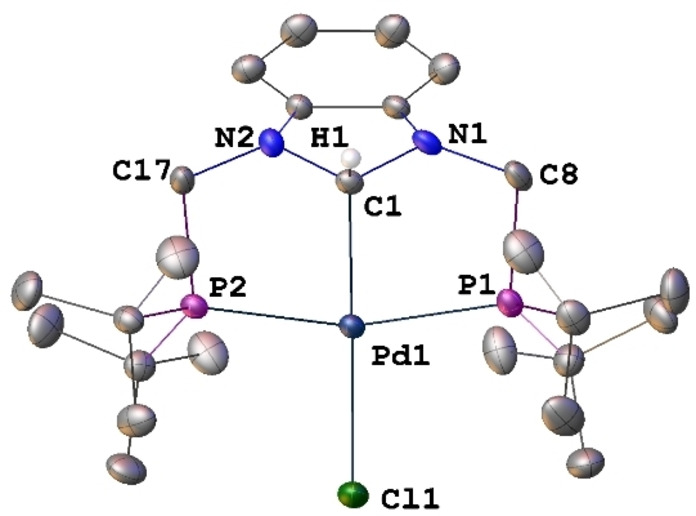
Structure of [PdCl(PC${}_{\mathrm{sp}{}^{3}}$ ^H^P)] (**3**) in **3** ⋅ 2 C_6_H_6_ with H atoms except PdC1−H1 omitted for clarity. Thermal ellipsoids are shown at 50 % probability. Selected bond lengths [Å] and angles [°]: Pd1−C1 2.086(2), Pd1−Cl1 2.4561(6), Pd1−P1 2.3288(6), Pd1−P2 2.3219(6), C1−N1 1.448(3), C1−N2 1.452(3); N1−C1−N2 102.19(17), N1−C1−Pd1 114.58(14), N2−C1−Pd1 115.19(14), C1−Pd1−Cl1 176.89(6), P1−Pd1−P2 163.34(2), C1−Pd1−P1 82.24(6), C1−Pd1−P2 82.13(6), P1−Pd1−Cl1 98.20(2), P2−Pd1−Cl1 97.72(2), N1−C1−N2 102.2(2).

When this reaction was performed in the presence of excess NEt_3_ for 2 h at room temperature, a yellow product **3** was isolated instead and fully characterized [Eq. (2) in Scheme 2]. Its ^1^H NMR spectrum contained a triplet at *δ* 6.95 (^3^ *J*(PH)=27.5 Hz), assigned to the N−CH−N proton that couples with the P nuclei which are magnetically equivalent in solution. The presence of a C${}_{\mathrm{sp}{}^{3}}$ −Pd bond in the neutral complex [PdCl(PC${}_{\mathrm{sp}{}^{3}}$ ^H^P)] (**3**) was confirmed by X‐ray diffraction analysis (Figures 2 and S18, Table S1), with a Pd−C1 bond length of 2.086(2) Å and a Pd−C1−H1 angle of 108.2°. Consistent with this, the five‐membered heterocycle mean plane makes an angle of 61.58° with the metal coordination plane. Structurally characterized palladium complexes with such a C−H‐bound N‐heterocyclic alkyl ligand are rare. The first such example was reported in 2013 with a Pd^II^ complex obtained by reaction between [Pd(PPh_3_)_4_] and an imidazolium chloride precursor containing two lateral diphenylphosphino‐substituted *o*‐phenylene linkers.^[62]^ The C${}_{\mathrm{sp}{}^{3}}$ −Pd bond distance of 2.0747(12) Å in this complex is very similar to that observed in **3**. In both cases, the chloride ligand contributes to the stabilization of the complex. It is noteworthy that the preference for alkyl versus NHC coordination has been rarely encountered in structurally characterized examples (Zr,^[93]^ Mn,^[94]^ Ni,[^95^, ^96^] Pd,[^62^, ^97^, ^98^] and Ir^[76]^).

**Scheme 3:**
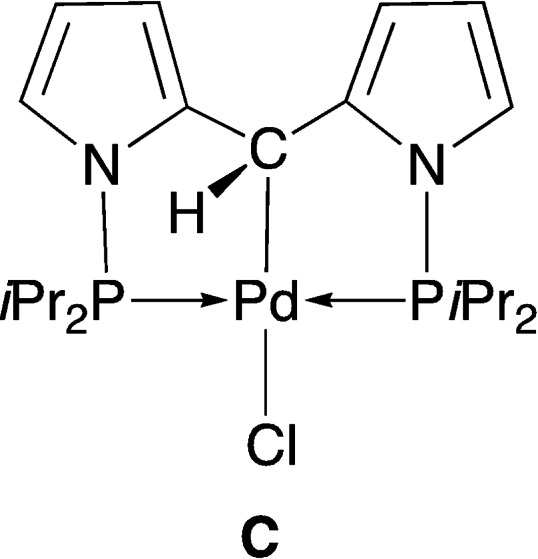
PC^alkyl^P Pd^II^ pincer system reported by Ozerov and co‐workers.^[80]^

The single C${}_{\mathrm{sp}{}^{3}}$ −H bond activation that occurred at the N−CH_2_−N moiety of **1** liberated HCl, the scavenging of which by NEt_3_ allowed the isolation of **3**. Indeed, reaction of isolated **3** with HCl in Et_2_O immediately produced **2**, strongly suggesting that **3** was an intermediate in the double C−H activation reaction leading from **1** to **2** [Eq. (3) in Scheme 2, see below]. This transformation thus occurs in two consecutive steps, where the first N−CH_2_−N hydrogen atom in **1** is formally eliminated as H^+^ whereas the remaining methine N−CH−N hydrogen in **3** behaves as a hydride, reacting with acids. In this context, it is interesting that with their PC^alkyl^P Pd^II^ pincer system **C** (Scheme 3), Ozerov and co‐workers also observed that the α‐CH moiety could not be deprotonated even by a strong base but behaved as a formal hydride donor.^[80]^

Subsequent work by Piers and Iluc and their co‐workers showed that in their respective PC^alkyl^P Ni^II^ and Pd^II^ pincer complexes, a non‐coordinating base and the presence of a suitable ligand are required for the deprotonation of the α‐CH to occur.[^79^, ^81^] The NiC${}_{\mathrm{sp}{}^{3}}$ −H hydrogen of **A** (Scheme 1) was found by Piers and co‐workers to be ambiphilic, and in the presence of KN(SiMe_3_)_2_ and PPh_3_ in THF, dehydrohalogenation of **A** afforded the pincer complex **B** with a Ni=C${}_{\mathrm{sp}{}^{2}}$ double bond.[^79^, ^83^] The reaction of Equation (3) is irreversible and no reaction was observed between **2** and NaH. Interestingly, in their study of the formation of PC^NHC^P pincer complexes through double C−H activation of a dihydroperimidine core by a Rh^I^ complex, Hill and McQueen suggested for the first C−H activation step the involvement of a putative σ‐2‐perimidinyl complex,^[76]^ which resembles our complex **3**. Very recently, C−H bond activation affording a PC^NHC^P Pd^II^ pincer complex from the six‐membered heterocyclic ligand in 1,3‐bis(diphenylphosphanylmethyl) hexahydropyrimidine was reported, but no intermediate was observed.^[88]^ A reaction pathway was proposed from DFT calculations, which involved a Pd^II^ hydride intermediate with an uncoordinated azolium moiety but the possibility of forming an intermediate similar to **3** was not discussed. It would be interesting to examine whether a reaction pathway similar to that established in our case (see Figure 4 below) could also be considered.

Furthermore, it was observed that 1,3‐dimethyl benzimidazoline, with two N−Me substituents instead of the N−CH_2_P*t*Bu_2_ groups of **1**, behaves as an “organic hydride” and reacts with acids with liberation of H_2_ and formation of the benzimidazolium cation.^[99]^ In our case, only the “second” C−H bond of **1** to be activated, that is, the C${}_{\mathrm{sp}{}^{3}}$ −H group in **3**, behaves as a hydride. All these observations highlight specific features associated with the formation of NHC‐containing pincer ligands and the unique reactivity of palladium in the chemistry of **1**.

The methine PdC${}_{\mathrm{sp}{}^{3}}$ −H group in **3** is highly reactive towards acids (see above), but not towards a basic reagent such as MeLi in benzene, where only chloride substitution and formation of the bis‐alkyl complex [PdMe(PC${}_{\mathrm{sp}{}^{3}}$ ^H^P)] (**4**) occurred [Eq. (4) in Scheme 2]. Similarly, the pincer complex **C** reacted with alkylating agents and only chloride substitution was observed.^[80]^ More recently, Iluc and co‐workers also found that the Pd^II^ chlorido complex supported by the PC${}_{\mathrm{sp}{}^{3}}$ ^H^P pincer ligand bis(2‐diisopropylphosphanyl)phenyl)methyl reacted with MeLi to give the corresponding methyl derivative, with retention of the alkyl pincer ligand.^[100]^

**Figure 3:**
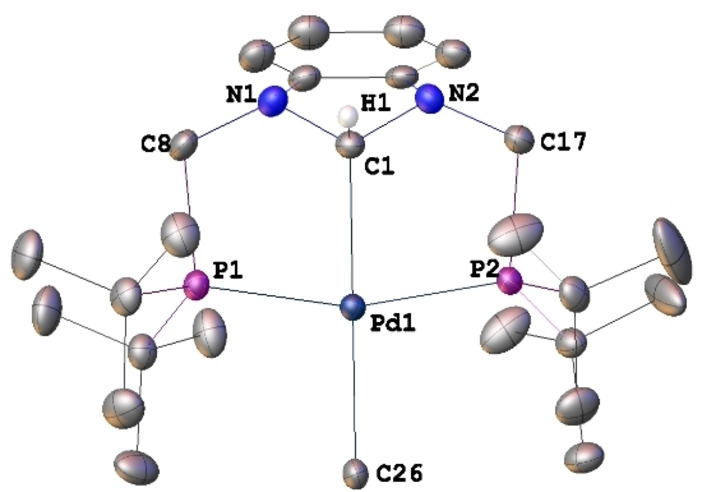
Structure of [PdMe(PC${}_{\mathrm{sp}{}^{3}}$ ^H^P)] (**4**) with H atoms except PdC1−H1 omitted for clarity. Thermal ellipsoids are shown at 50 % probability. Selected bond lengths [Å] and angles [°]: Pd1−C1 2.1323(15), Pd1−C26 2.1884(15), Pd1−P1 2.2939(4), Pd1−P2 2.2990(4), C1−N1 1.469(2), C1−N2 1.4721(19); N1−C1−N2 100.81(12), N1−C1−Pd1 115.20(10), N2−C1−Pd1 114.67(10), C1−Pd1−C26 178.46(6), P1−Pd1−P2 162.28(2), C1−Pd1−P1 81.66(4), C1−Pd1−P2 82.07(4), P1−Pd1−C26 98.66(4), P2−Pd1−C26 97.79(4), N1−C1−N2 100.81(12).

The crystal structure of **4** (Figure 3) is similar to that of **3** with a Pd−C1 bond length of 2.1323(15) Å, longer than in **3** owing to the larger *trans* influence of the methyl group compared to chloride. The five‐membered heterocycle mean plane makes an angle of 65.18° with the metal coordination plane. A similar metal alkylation occurred when the Pd^II^ complex analogous to **A** was reacted with MeLi to give a Pd^II^−Me complex with retention of the PdC${}_{\mathrm{sp}{}^{3}}$ −H bond.^[100]^ No reaction was observed at room temperature between **4** and ethylene (^1^H NMR monitoring in a Young tube). Nucleophilic substitution of the chloride ligand in **3** also occurred with NaN_3_ in acetone, at room temperature in the presence of [15]crown‐5, to afford [PdN_3_(PC${}_{\mathrm{sp}{}^{3}}$ ^H^P)] (**5**) [Eq. (5)], which was characterized by X‐ray diffraction (Table S2 and Figure S20) and displays a bent azide ligand (Pd1−N3−N4 120.3(2)°). Photochemical irradiation (*λ*=365 or 254 nm, 4 W) of this complex did not result in liberation of N_2_. When a benzene solution of **3** was exposed to air, the solution turned purple within seconds, but the corresponding highly soluble compound could not be characterized. The only peak in its mass spectrum that could be assigned corresponded to **3**‐H. This reaction was accompanied by the formation of **2**, probably as a result of partial decomposition and liberation of HCl, that would readily protonate unreacted **3**. Similarly, a purple colour developed when a benzene solution of **5** was exposed to air. In contrast, a solution of the pincer complex **4** in benzene or pentane was air‐stable for at least 0.5 h, although the methyl ligand renders this complex more electron‐rich. In attempts to better characterize the purple compound resulting from exposure of **3** to air, we prepared the analogue of **3** from the diphenylphosphino‐substituted ligand **1** ${}^{\mathrm{Ph}{}_{2}}$ ,^[92]^ that was expected to reduce the solubility of the complex. As with **1**, C${}_{\mathrm{sp}{}^{3}}$ −H bond activation occurred upon reaction with [PdCl_2_(cod)] to give **3** ${}^{\mathrm{Ph}{}_{2}}$ . However, this complex did not change colour upon exposure of its solution to air, which highlights the critical role of the strong electron‐donating *t*Bu substituents in **3** which make the metal more electron‐rich and thus much more air‐sensitive.

**Figure 4:**
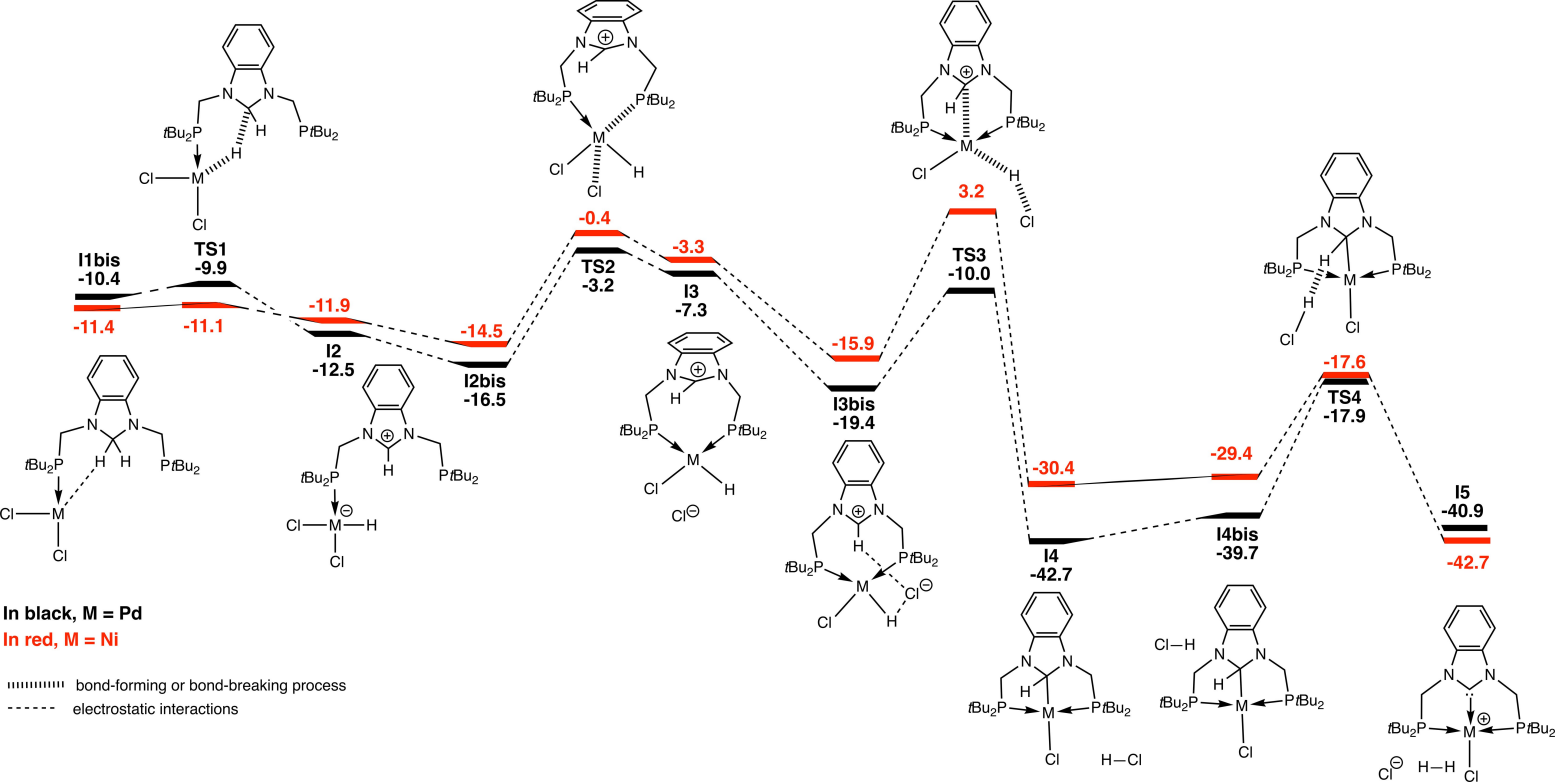
Mechanism of the CH_2_ double activation with energies in kcal ⋅ mol^−1^.

**Figure 5:**
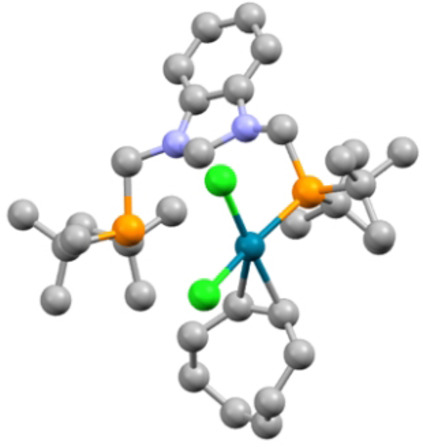
Computed structure of the Pd^II^ intermediate **I1**, hydrogen atoms are omitted for clarity.

We decided to explore the mechanism of the CH_2_ double activation by DFT methods. Starting from the adduct **I1bis**, the successive steps are shown in Figure 4. This adduct results from the coordination of **1** on [PdCl_2_(cod)] to give first an intermediate **I1** in which both the cod ligand and **1** are monodentate (Figure 5).

This step is exergonic (Δ*G*=−8.6 kcal mol^−1^ for **I1**) and results in several conformers from which different reaction paths are conceivable. Three mechanisms were tested for the CH_2_ double activation reaction. The first one starts from **I1bis** (Figure 4, Δ*G*=−10.4 kcal ⋅ mol^−1^) in which the halides are in cis position, the fourth site of the palladium square planar coordination sphere being occupied by an agostic interaction (Pd−H distance of 1.732 Å) involving one hydrogen atom of the CH_2_ group, which strongly weakens the C−H bond (1.246 Å). This step opens the way to the hydride transfer yielding an imidazolium zwitterionic complex **I2** through a low transition state **TS1** (Δ*G*=−9.9 kcal ⋅ mol^−1^; Figure 4). This leads to **I2bis** through **I2**, both structures being rotamers around a C−P bond (only one structure is shown in Figure 4). The next step is the substitution of one chloride ligand by the second phosphorus donor atom with a barrier of 14.1 kcal ⋅ mol^−1^ (**TS2‐I2bis**). The intermediate **I3** evolves to give **I3bis** in which the free chloride anion interacts with the Pd‐bound hydride. This interaction becomes stronger and **TS3** is associated with a barrier of 9.4 kcal ⋅ mol^−1^ (**TS3‐I3bis**) and results in the removal of this ligand as a proton, in a formal redox reaction. Thus, the electronic doublet ensuring the Pd−H bond is transferred to the imidazolium moiety that is reduced to an imidazolate. This step is mediated by the palladium and a Pd−C bond is formed with the imidazolate. In the final step, the HCl fully liberated (**I4** to **I4bis**) attacks the N−C*H−*N hydrogen to give the final product **I5** with liberation of H_2_. Consistent with the key role of HCl in this step, its trapping by a base stops the reaction at the intermediate stage **I4**. This is consistent with the relatively high barrier (**TS4‐I4**) of 24.8 kcal ⋅ mol^−1^ associated to **TS4** compared to the previous steps: the amine traps the acid before it reacts. It should be noticed that not only the barrier associated to **TS4** is high but the reaction is also endergonic, **I5** (Δ*G*=−40.9 kcal ⋅ mol^−1^) being less stable than **I4** (Δ*G*=−42.7 kcal ⋅ mol^−1^). The overall exergonicity of the reaction is ensured by the H_2_ departure that stabilizes the system by −4.6 kcal ⋅ mol^−1^ (Δ*G*=−45.5 kcal ⋅ mol^−1^ for the computed structure of **2**, see Table S3).

**Figure 6:**
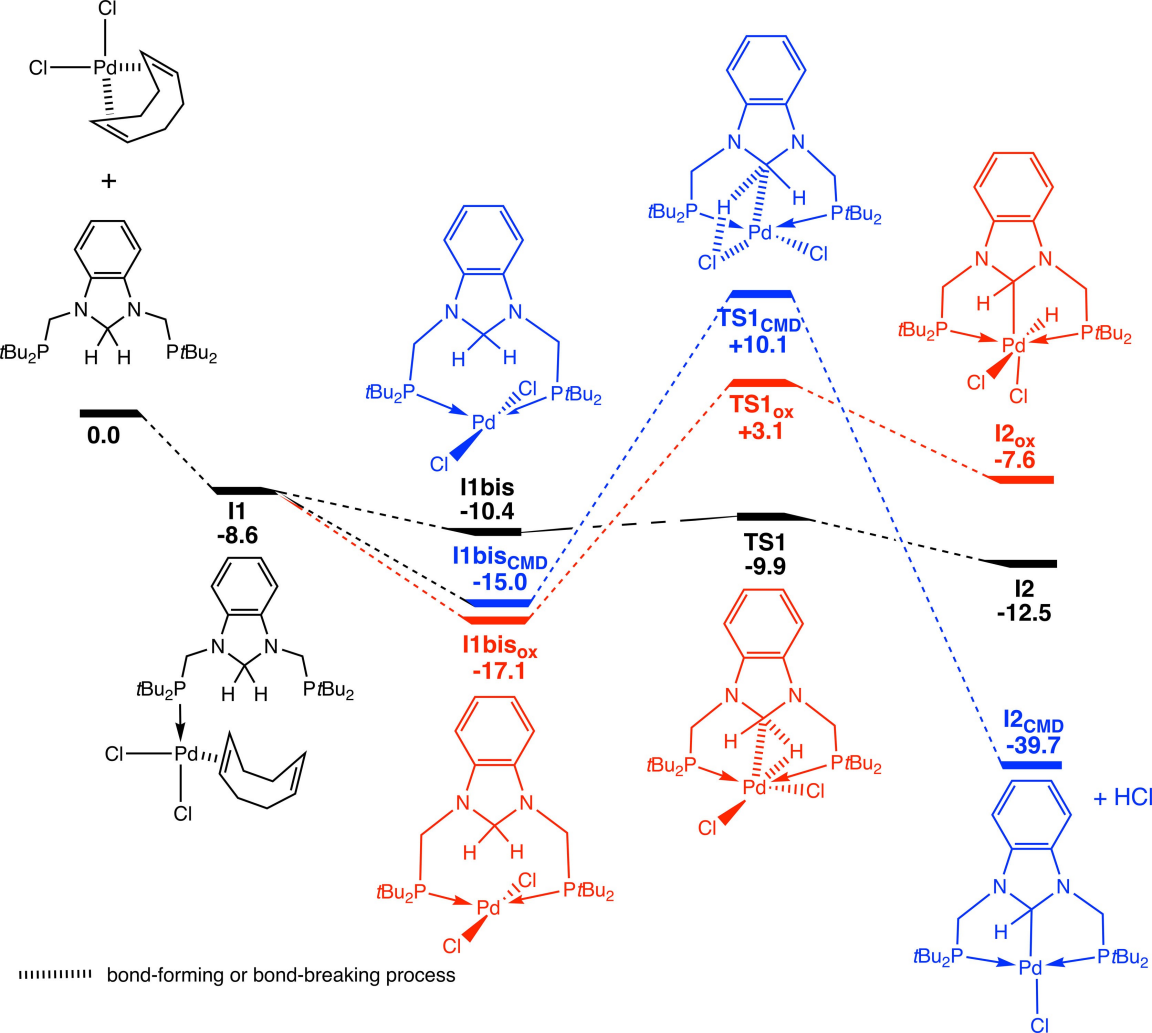
Alternative mechanisms for the CH_2_ double activation with energies in kcal ⋅ mol^−1^. the mechanism proposed in Figure 4 is in black, the mechanism for the oxidative addition in red and the CMD mechanism is in blue.

We then investigated two alternative mechanisms for CH_2_ activation with palladium. We considered the formal oxidative addition of one CH bond on the palladium(II) (path thereafter denoted as OX) which involves metal insertion into a C−H bond of the CH_2_ group to give formally a Pd^IV^ species. We also explored the feasibility of a cyclometallation–deprotonation mechanism (path hereafter denoted CMD), in which a C−H atom is eliminated as HCl with the chloride ligand.^[101]^ In Figure 6, we present the energetics of the first step of these two pathways and compare them with those described in Figure 4. **I2**_CMD_ is equivalent to **I4**bis and **I2**_ox_ will undergo a reductive elimination also to give an equivalent of **I4bis**. The final step of these two alternative mechanisms will be the same as that of Figure 4 starting from **I4bis**. Most importantly, the energy associated with **TS1** is higher for the OX and CMD mechanisms than for that of Figure 4. These transition states (**TS1_OX_** and **TS1_CMD_**) are even higher in energy than all those computed for the first proposed path, making them non‐competitive. Consequently, we can exclude these mechanisms (OX and CMD) for the double CH_2_ bond activation with palladium under our reaction conditions. Note that reactions following a typical CMD mechanism usually involve a carboxylate ligand which remains coordinated to the metal at the end of the reaction, in contrast to the chloride ligand that dissociates from the metal centre.

**Scheme 4:**
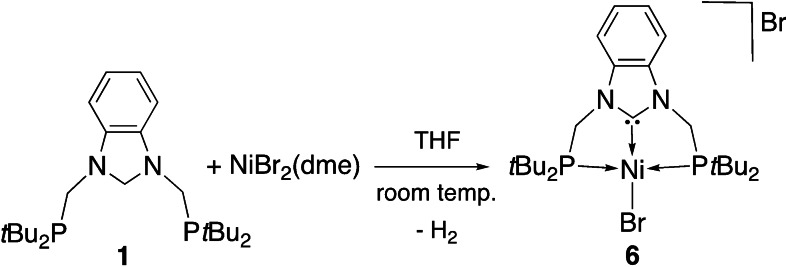
Synthesis of the NHC pincer complex [NiBr(PC^NHC^P)]Br (**6**).

**Figure 7:**
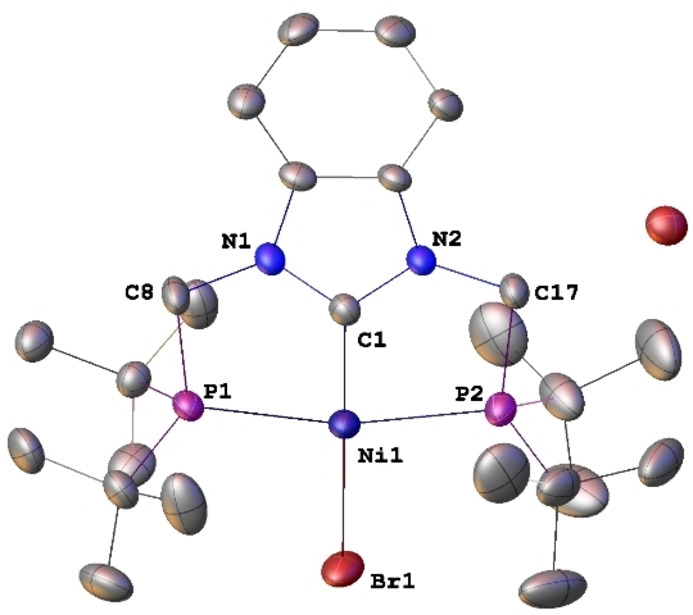
Structure of the NHC pincer complex [NiBr(PC^NHC^P)]Br (**6**) with H atoms omitted for clarity. Thermal ellipsoids are shown at 50 % probability. Selected bond lengths [Å] and angles [°]: Ni1−C1 1.840(3), Ni1−Br1 2.3224(5), Ni1−P1 2.2610(9), Ni1−P2 2.2588(9), C1−N1 1.348(4), C1−N2 1.364(4); N1−C1−N2 106.7(3), N1−C1−Ni1 126.8(2), N2−C1−Ni1 126.6(2), C1−Ni1−Br1 179.61(10), P1−Ni1−P2 166.68(4), C1−Ni1−P1 83.25(10), C1−Ni1−P2 83.46(10), P1−Ni1−Br1 96.43(3), P2−Ni1−Br1 96.86(3).

For comparison with the reaction of [Eq. (1)], we examined the reaction of **1** with [NiBr_2_(dme)] in THF at room temperature and observed a very fast reaction leading directly to the NHC pincer complex [NiBr(PC^NHC^P)]Br (**6**) (Scheme 4). It was fully characterized and shows a square‐planar environment for Ni^II^, similar to that of Pd^II^ in complex **2** (Figure 7).

**Scheme 5:**
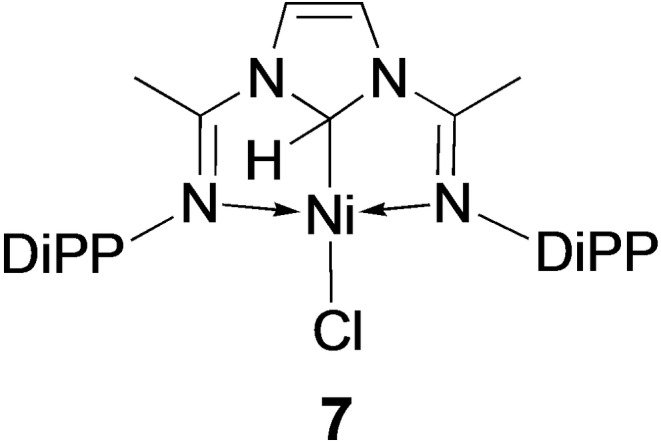
The Ni^II^ N^imine^C${}_{\mathrm{sp}{}^{3}}$ ^H^N^imine^ pincer complex **7**.^[95]^

With Ni^II^, the reactions leading to the double C${}_{\mathrm{sp}{}^{3}}$ −H bond activation of the N−CH_2_−N moiety of **1** were too rapid to allow the observation or isolation of an intermediate with a covalent C${}_{\mathrm{sp}{}^{3}}$ −Ni bond, even in the presence of NEt_3_ (in CD_2_Cl_2_ or C_6_D_6_). In contrast, the Pd^II^ intermediate **4** was readily isolated (Scheme 2). Interestingly, a related Ni^II^ complex **7** has been recently characterized (Scheme 5), which displays a diimine alkyl pincer ligand, although the alkyl function did not result from activation by Ni^II^ of a neutral N−CH_2_−N moiety but from oxidative‐addition of the imidazolium C2−H bond across Ni^0^.^[95]^ The relative stability of **7**, and its lack of evolution to give a N^imine^C^NHC^N^imine^ nickel hydride,^[97]^ were shown by computational methods to result from geometric constraints brought about by the rigidity of the pincer ligand. Interestingly, in a complex analogous to **7** but containing a six‐membered 1,4,5,6‐tetrahydropyrimidine ring, the NiC${}_{\mathrm{sp}{}^{3}}$ −H bond was deprotonated by KHMDS in toluene to give the corresponding NHC pincer complex,^[63]^ similarly to observations made in Scheme 1 (**A**→**B**), but in contrast to the behaviour of the PdC${}_{\mathrm{sp}{}^{3}}$ −H bond in **3**.

For comparison, we also computed the reaction mechanism with Ni and the overall mechanism was found to be similar to that with Pd. However, significant differences were observed in the energies involved. The first steps are very similar in energy, the complexation of the Ni^II^ centre by the organic ligand is slightly stronger (−11.4 kcal ⋅ mol^−1^) than that of Pd^II^ (−10.4 kcal ⋅ mol^−1^, Figure 4). The barrier for the hydride transfer (**I1bis** to **TS1**) is slightly higher with Pd than with Ni (0.5 and 0.3 kcal ⋅ mol^−1^, respectively). The trend is reversed for the next two steps, **TS2** and **TS3**, since the barriers are higher for Ni (14.1 and 19.1 kcal ⋅ mol^−1^) than for Pd (13.2 and 9.4 kcal ⋅ mol^−1^), respectively. However, the energy barrier for the last step (**I4** to **TS4**) is significantly higher for Pd (24.8 kcal ⋅ mol^−1^) than for Ni (12.8 kcal ⋅ mol^−1^). This step is rate determining in the case of palladium but not of nickel, for which it is associated to **TS3**. These values explain the experimental findings. The highest barrier for palladium is larger than that for nickel, consequently the reaction will be faster for the latter. The small energy barrier associated to **TS4** for nickel explains the failure to trap the hydride intermediate: the reaction is too fast, and the addition of an amine cannot prevent the acid of reacting. On the contrary, the high barrier in the case of palladium allows trapping of the intermediate. Another difference is the endergonicity of the reaction with palladium, **I5** is less stable than **I4bis**, the exergonicity of the reaction being ensured by the departure of the H_2_ molecule. This is not the case for nickel where **I5** is more stable than **I4bis**.

With diphosphines such as **1** as precursors to PC^NHC^P pincer systems, coordination of the P donors to the MX_2_ unit is most likely to occur first, and this brings the CH_2_ group to be activated in closer proximity to the metal M. This step could be further assisted by an additional interaction between a N atom of the heterocycle and the metal centre,^[86]^ which facilitates the oxidative addition of the first C${}_{\mathrm{sp}{}^{3}}$ −H bond to be activated, resulting in a C${}_{\mathrm{sp}{}^{3}}$ −M bond. This step would be followed by reductive‐elimination of HX, or σ‐bond metathesis between a C−H and a M−X bond, or by dissociation of a halide followed by anion‐promoted deprotonation. These possibilities have been examined recently.^[19]^

In addition to providing new insights into the double geminal activation of C${}_{\mathrm{sp}{}^{3}}$ −H bonds, this work illustrates major differences between pincer ligands bearing a NHC donor in the bridgehead position but different side arms (phosphines vs. imines) and for a given pincer ligand, the differences between the metal centres, Ni being generally far more reactive than Pd, although the latter sometimes allows, like in this study, the isolation of intermediates that shed light on the course of important and catalytically relevant reactions, such as C${}_{\mathrm{sp}{}^{3}}$ −H bond activation and NHC formation.

## Conclusion

Although it was known that metal‐promoted double C${}_{\mathrm{sp}{}^{3}}$ −H activation can lead to metal carbene complexes,[^5^, ^69^] this attractive and straightforward protocol has, to the best of our knowledge, been applied here for the first time to the N−CH_2_−N moiety of an imidazole‐type heterocycle, directly affording a coordinated NHC ligand under mild conditions. Such facile, room‐temperature metal‐promoted access to highly popular NHC‐metal complexes offers considerable synthetic potential as readily accessible and stable Ni^II^ or Pd^II^ metal reagents were used. Thanks to their versatile chemistry and flexible coordination sphere, both Ni^II^ or Pd^II^ complexes were competent to perform the double C${}_{\mathrm{sp}{}^{3}}$ −H bond activation, but the reactions were too fast with Ni^II^ to identify any intermediate. With Pd^II^, in contrast, a key alkyl intermediate resulting from single C${}_{\mathrm{sp}{}^{3}}$ −H bond activation was isolated and fully characterized. As discussed in this paper, several examples have shown that the first step of the C${}_{\mathrm{sp}{}^{3}}$ −H bond activation of a N−CH_2_−N moiety involves the formation of a metal alkyl complex. The pathways leading to the activation of the resulting methine C${}_{\mathrm{sp}{}^{3}}$ H−M group have been generally less discussed, probably because such intermediates have more rarely been isolated. Our combined theoretical and experimental study has provided new insights into the mechanism of the double geminal activation of the C${}_{\mathrm{sp}{}^{3}}$ −H bonds of a N−CH_2_−N moiety and shown that the properties and reactivity of the C${}_{\mathrm{sp}{}^{3}}$ H−M bond generated in the first step of the reaction depend very much on subtle effects. Overall, the double C${}_{\mathrm{sp}{}^{3}}$ −H activation of the N−CH_2_−N hydrogen atoms of **1** can be formally viewed as the metal‐ and chelate‐assisted successive elimination of H^+^ and H^−^. Our results extend to PC^NHC^P chemistry the double C${}_{\mathrm{sp}{}^{3}}$ −H activation approach leading to PC${}_{\mathrm{sp}{}^{2}}$ P pincer complexes for which the first C−H activation step occurs by oxidative addition or electrophilic activation, and the subsequent activation of the α‐methine group by α‐hydride elimination, hydride or proton abstraction.

Future work should explore the scope of the protocols described in this work to access diverse NHC pincer complexes. The associated advantages of starting directly from one of the many neutral heterocyclic candidates containing a N−CH_2_−N moiety, without the need to prepare an azolium derivative or a free carbene, and of using air stable and readily available metal precursors, will facilitate the study of their reactivity.

Deposition Numbers 2115914 (**1**), 2115915 (**2⋅**CH_2_Cl_2_), 2115916 (**3⋅**2 C_6_H_6_), 2115917 (**4**), 2115918 (**5**) and 2115919 (**6**) contain the supplementary crystallographic data for this paper. These data are provided free of charge by the joint Cambridge Crystallographic Data Centre and Fachinformationszentrum Karlsruhe Access Structures service.

## Conflict of interest

The authors declare no conflict of interest.

1

## Supporting information

As a service to our authors and readers, this journal provides supporting information supplied by the authors. Such materials are peer reviewed and may be re‐organized for online delivery, but are not copy‐edited or typeset. Technical support issues arising from supporting information (other than missing files) should be addressed to the authors.

Supporting Information

## Data Availability

The data that support the findings of this study are available in the supplementary material of this article.
