# Supplementary material for: Experimental and Theoretical Study of NiII‐ and PdII‐Promoted Double Geminal C(sp3)−H Bond Activation Providing Facile Access to NHC Pincer Complexes: Isolated Intermediates and Mechanism
Source: Chemistry. 2022 Jun 13;28(41):e202200507. doi: 10.1002/chem.202200507 (PMC9401054; doi:10.1002/chem.202200507)
Supplement: Supplementary file 1 — Supporting Information [file CHEM-28-0-s001.pdf]

Chemistry–A European Journal

Supporting Information

Experimental and Theoretical Study of Ni^{II}- and Pd^{II}-Promoted Double Geminal $C(sp^3)$–H Bond Activation Providing Facile Access to NHC Pincer Complexes: Isolated Intermediates and Mechanism

Fengkai He, Christophe Gourlaouen, Huan Pang,* and Pierre Braunstein*

Table of Contents

Experimental Details 3

1. Synthesis and Characterization 3

1.1. General methods. 3

1.2. Preparation of di-tert-butylphosphine oxide (L1). 4

1.3. Preparation of di-tert-butyl(hydroxymethyl)phosphine oxide (L2). 4

1.4. Preparation of (di-tert-butylphosphoryl)methyl 4-methylbenzenesulfonate (L3). 5

1.5. Preparation of ((1H-benzo[d]imidazol-1-yl)methyl)di-tert-butylphosphine oxide (L4). 5

1.6. Preparation of 1,3-bis((di-tert-butylphosphoryl)methyl)-1H-benzo[d]imidazol-3-ium 4-methylbenzenesulfonate (L5). 6

1.7. Preparation of 1,3-bis((di-tert-butylphosphanyl)methyl)-2,3-dihydro-1H-benzo[d] imidazole (1). 6

1.8. Preparation of complex 2. 7

1.9. Preparation of complex 3. 7

1.10. Preparation of complex 4. 8

1.11. Preparation of complex 5. 9

1.12. Preparation of complex 6. 10

1.13. Reaction of complex 3 with HCl in Et_2O. 10

1.14. Reaction of complex 3 with air. 10

1.15. Reaction of complex 5 with air. 11

1.16. NMR spectra. 12

2. X-ray crystallography 20

2.1. General methods. 20

2.2. Summary of crystal data. 20

2.3. Crystal structure of the ligand 1. 23

2.4. Crystal structure of $[PdCl(PC^{NHC}P)]Cl\cdot CH_2Cl_2$ ($2\cdot CH_2Cl_2$). 24

2.5. Crystal structure of $[PdCl(PC^{H}P)]\cdot 2C_6H_6$ ($3\cdot 2C_6H_6$). 25

2.6. Crystal structure of $[PdN_3(PC^{H}P)]$ (5) 25

3. Computational details 26

References 28

Experimental Details

1. Synthesis and Characterization

1.1. General methods.

All manipulations involving organometallics were performed under nitrogen or argon in a Braun glove-box or using standard Schlenk techniques. All solvents were dried using standard methods and distilled under nitrogen prior use. The starting materials **L1**,[1,2] **L3**,[3] L4[3] and **L5**[3] were prepared according to the literature and the reactions leading to the new diphosphine **1** are summarized in Scheme S1 (below).

^1H, $^{13}C\{^1H\}$, and $^{31}P\{^1H\}$ NMR spectra were recorded on Bruker spectrometers (AVANCE III – 400 MHz or AVANCE I – 500 MHz equipped with a cryogenic probe). Downfield shifts are reported in ppm as positive and referenced using signals of the residual proton solvent (^1H), the solvent (^{13}C) or externally (^{31}P). All NMR spectra were measured at 298 K, unless otherwise specified. Mass spectra were recorded on Bruker micrOTOF II. Elemental analyses were performed by the "Service de microanalyses", Université de Strasbourg.

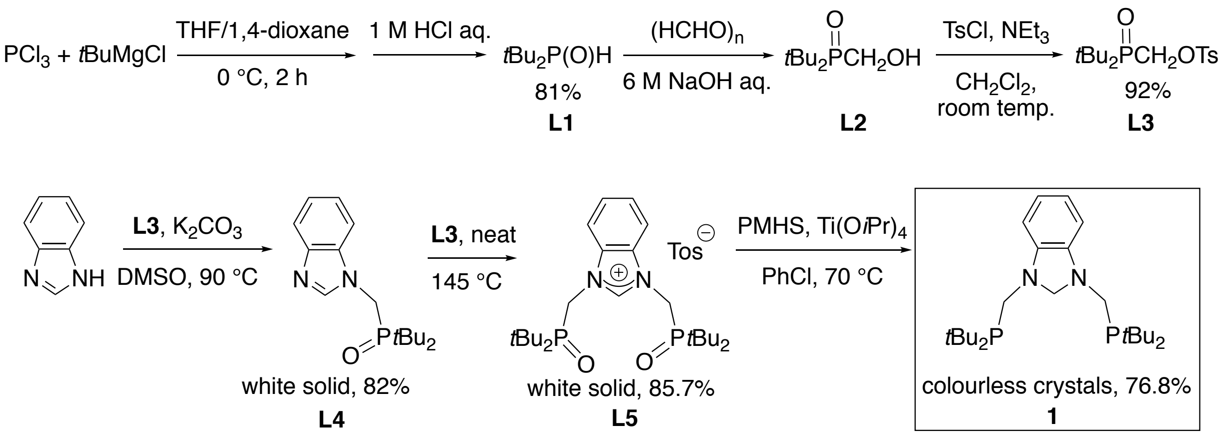

Scheme S1. Synthesis of the diphosphine ligand **1**

1.2. Preparation of di-*tert*-butylphosphine oxide (L1).

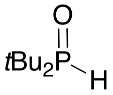
L1

A solution of *t*BuCl (20.00 g, 146.0 mmol) in THF (180 ml) was added dropwise to a suspension of Mg turnings (3.55 g, 145.9 mmol) in THF (20 ml) under N_2 atmosphere to form the Grignard reagent. At 0 °C, the fresh Grignard reagent was added dropwise into the mixture of PCl_3 (5.00 g, 36.5 mmol) in THF (50 ml) and 1,4-dioxane (20 ml), and white $MgCl_2$ was formed rapidly. ^{31}P NMR spectroscopy was used to monitor the reaction. After it was finished, 200 ml HCl solution (0.1 M) was added dropwise to the mixture. THF was removed, CH_2Cl_2 was added to extract the product, and the organic layer was dried over $MgSO_4$ and filtered. The solution was concentrated, the crude product was purified by column chromatography (CH_2Cl_2/MeOH = 20:1) and the pure white solid was isolated in 76% yield (4.50 g). ^{1}H NMR (400 MHz, $CDCl_3$): δ 5.87 (d, ^1J(PH) = 426.7 Hz, 1H), 1.08 (d, ^3J(PH) = 15.1 Hz, 18H). ^{13}C NMR (101 MHz, $CDCl_3$): δ 33.55 (d, ^1J(CP) = 59.1 Hz), 25.49 (s, CH_3). ^{31}P NMR (162 MHz, $CDCl_3$): δ 66.51.

1.3. Preparation of di-*tert*-butyl(hydroxymethyl)phosphine oxide (L2).

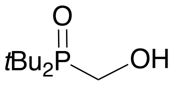
L2

Compound **L1** (10.00 g, 61.6 mmol) and paraformaldehyde (5.55 g, 184.8 mmol) were added to a NaOH solution (100 ml, 6 M), the mixture was heated to 70 °C for 12 h and the reaction was monitored by ^{31}P NMR spectroscopy. At the end of the reaction, the mixture was cooled to room temperature, and HCl (600 ml, 1 M) was added to neutralize the excess NaOH. CH_2Cl_2 was used to extract the product from the solution and the organic layer was filtered over $MgSO_4$. The crude product was obtained by concentration of the solution and purified by column chromatography (CH_2Cl_2/MeOH = 20:1) and isolated as a white solid in 87% yield. ^{1}H NMR (400 MHz, $CDCl_3$): δ 4.10 (br s, 1H, OH), 3.99 (s, 2H, CH_2), 1.28 (d, ^2J(HP) = 13.2 Hz, 18H, CH_3). ^{31}P NMR (162 MHz, $CDCl_3$): δ 58.28.

1.4. Preparation of (di-*tert*-butylphosphoryl)methyl 4-methylbenzenesulfonate (L3).

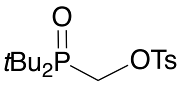

L3

A solution of TsCl (1.58 g, 15.6 mmol) in CH_2Cl_2 (30 ml) was added dropwise to a solution of **2** (2.50 g, 13.0 mmol) and NEt_3 (3.00 g, 15.6 mmol) in CH_2Cl_2 (20 ml) over 30 min. After the mixture was further stirred for 2 h at room temperature, water (50 ml) was added and CH_2Cl_2 was used to extract compound **L3**. The organic layer was dried over $MgSO_4$ and the crude product was obtained by concentration of the filtered solution. Product **L3** was isolated as a pure white solid after purification by column chromatography (CH_2Cl_2/MeOH = 20:1) in 88.8% yield (4.00 g). ^1H NMR (400 MHz, $CDCl_3$): δ 7.79 (d, ^{3+5}J(HH) = 8.4 Hz, 2H, aromatic), 7.37 (d, ^{3+5}J(HH) = 8.4 Hz, 2H, aromatic), 4.26 (d, ^2J(HP) = 6.9 Hz, 2H, CH_2), 2.46 (s, 3H, CH_3, tosyl), 1.27 (d, ^3J(HP) = 13.9 Hz, 18H, CH_3). ^{31}P NMR (162 MHz, $CDCl_3$): δ 55.87.

1.5. Preparation of ((1H-benzo[d]imidazol-1-yl)methyl)di-*tert*-butylphosphine oxide (L4).

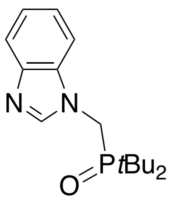

L4

A mixture of benzimidazole (2.40 g, 20.3 mmol), K_2CO_3 (19.60 g, 142.2 mmol) and **3** (10.60 g, 30.5 mmol) in DMSO (100 ml) was stirred for 24 h at 110 °C. After the solvent was removed under reduced pressure, CH_2Cl_2 was added to dissolve the residue and the organic layer was washed with water and dried over $MgSO_4$. The crude product was obtained by concentration, and purification by washing with Et_2O afforded **L4 a**s a pure white solid in 87.5% yield (5.20 g). ^1H NMR (400 MHz, $CDCl_3$): δ 8.53 (s, 1H, NCH), 7.81 (d, *J* = 7.7 Hz, 1H), 7.47 (d, *J* = 7.7 Hz, 1H), 7.34 (t, *J* = 7.1 Hz, 1H), 7.29 (t, *J* = 7.1 Hz, 1H), 4.53 (d, ^2J(HP) = 4.4 Hz, 2H), 1.27 (d, ^3J(HP) = 13.6 Hz, 18H, CH_3). ^{31}P NMR (162 MHz, $CDCl_3$): δ 55.82.

1.6. Preparation of 1,3-bis((di-*tert*-butylphosphoryl)methyl)-1H-benzo[d]imidazol-3-ium 4-methylbenzenesulfonate (L5).

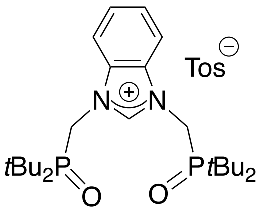

L5

A mixture of neat **L3** (3.44 g, 9.92 mmol) and **L4** (1.45 g, 4.96 mmol) was heated to 145 °C for 48 h. After it was cooled, the mixture was dissolved in CH_2Cl_2 and addition of toluene/Et_2O (v/v = 20:1) precipitated a white solid, which was collected by filtration. The solution was concentrated to get a second crop of the white solid, which was collected by filtration and washed with toluene. Product **L5** was obtained as white solid in a total yield of 82% (2.60 g). ^{1}H NMR (400 MHz, $CDCl_3$): δ 10.63 (s, 1H, NCH), 8.10 (dd, *J* = 6.4, 3.2 Hz, 2H, aromatic), 7.69 (d, *J* = 8.0 Hz, 2H, tosylate), 7.58 (dd, *J* = 6.4, 3.1 Hz, 2H, aromatic), 7.09 (d, *J* = 8.0 Hz, 2H, tosylate), 5.10 (d, ^2J(HP) = 3.5 Hz, 4H, PCH_2), 2.32 (s, 3H, tosylate Me), 1.32 (d, ^3J(HP) = 14.1 Hz, 36H, CH_3). ^{31}P NMR (162 MHz, $CDCl_3$): δ 56.67.

1.7. Preparation of 1,3-bis((di-*tert*-butylphosphanyl)methyl)-2,3-dihydro-1H-benzo[d] imidazole (1).

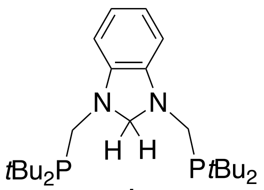

1

A mixture of **L5** (1.40 g, 2.19 mmol), polymethylhydrosiloxane (PMHS) (1.9 ml) and [Ti(O^{i}Pr)$_4$] (2.0 ml) in PhCl was stirred at 70 °C for 12 h under N_2 atmosphere. The solvent was removed under reduced pressure and pentane was added. The solution was filtered through Celite and concentrated. Colourless crystals were obtained from pentane below -30 °C (0.73 g, in 76.8% yield). ^{1}H NMR (500 MHz, C_6D_6): δ 6.86 (dd, *J* = 5.5, 3.1 Hz, 2H, aromatic), 6.65 (dd, *J* = 5.5, 3.2 Hz, 2H, aromatic), 4.95 (s, 2H, NCH_2), 3.26 (d, ^2J(HP) = 4.1 Hz, 4H, PCH_2), 1.10 (d, ^3J(HP) = 10.8 Hz, 36H, CH_3). ^{13}C NMR (126 MHz, C_6D_6) (assignments from DEPT experiments): δ 143.61 (dd, *J* = 5.2, 1.8 Hz, N*C*C), 119.56 (s, aromatic), 106.68 (s, aromatic), 78.85 (t, ^3J(C,P) = 9.8 Hz, NCH_2N), 44.02 (d, ^1J(CP) = 16.0 Hz, CH_2P), 31.21 (d, ^1J = 22.4 Hz, CP), 29.71 (d, ^2J(CP) = 13.0 Hz, CH_3). ^{31}P NMR (203 MHz, C_6D_6): δ 11.67. ESI-MS: *m/z*

$[C_{25}H_{46}N_2P_2+Na]^+$ (*M* + 23)$^+$ 459.30. Anal. calcd. for $C_{25}H_{46}N_2P_2$: C, 68.78; H, 10.62; N, 6.42%; found: C, 68.40; H, 10.63; N, 6.38%.

The reactions leading to diphosphine **1** are summarized above in Scheme S1.

1.8. Preparation of complex 2.

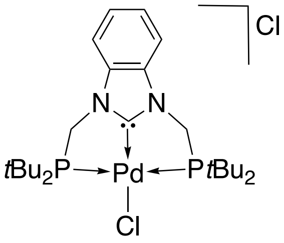

2

A mixture of **1** (0.100 g, 0.229 mmol) and $[PdCl_2(cod)]$ (0.0654 g, 0.229 mmol) in CH_2Cl_2 (20 ml) was stirred overnight at room temperature. The volatiles were removed under reduced pressure and THF (10 ml) was added to precipitate a white solid which was collected by filtration in 70% yield (0.097 g). X-ray quality crystals were obtained by slow diffusion of pentane into a CH_2Cl_2 solution. ^{1}H NMR (400 MHz, $CDCl_3$): δ 8.16 (dd, *J* = 6.1, 3.2 Hz, 2H, aromatic), 7.43 (dd, *J* = 6.1, 3.1 Hz, 2H, aromatic), 5.10 (vt, ^{2+4}J(HP) = 2.3 Hz, 4H, PCH_2), 1.55 (t, ^{3+5}J(HP) = 7.9 Hz, 36H, CH_3). ^{13}C NMR (126 MHz, $CDCl_3$) (assignments from DEPT experiments): δ 176.13 (t, ^2J(CP) = 2.9 Hz, NCN), 133.19 (t, ^{3+4}J(CP) = 4.8 Hz, N*C*C), 126.17 (s, aromatic), 114.35 (s, aromatic), 44.53 (t, ^{1+3}J(CP) = 11.3 Hz, CH_2P), 37.47 (t, ^{1+3}J(CP) = 7.4 Hz, CP), 29.35 (t, ^{2+4}J(CP) = 2.8 Hz, CH_3). ^{31}P NMR (162 MHz, $CDCl_3$): δ 81.39. ESI-MS: *m/z* $[C_{25}H_{44}N_2P_2PdCl]^+$ (*M*)$^+$ 575.17. Anal. calcd. for $[C_{25}H_{44}N_2P_2PdCl_2 + CH_2Cl_2]$: C, 44.81; H, 6.65; N, 4.02%; found: C, 43.95; H, 6.95; N, 4.12%. Despite several attempts, no better elemental analyses could be obtained.

1.9. Preparation of complex 3.

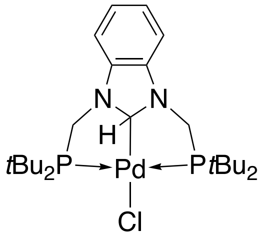

3

A mixture of **1** (0.100 g, 0.229 mmol), $[PdCl_2(cod)]$ (0.0654 g, 0.229 mmol) and NEt_3 (0.100 g, 0.988 mmol) in CH_2Cl_2 (20 ml) was stirred at room temperature for 2 h. After the volatiles were removed under reduced pressure, the residue was washed with pentane. It was dissolved in THF and the yellow solution was filtered and concentrated, affording a yellow solid which was redissolved in toluene (to remove traces of **2**), and the yellow solution was filtered. Slow evaporation afforded yellow crystals of highly air sensitive **3** in 34.8%

yield. ^{1}H NMR (600 MHz, C_6D_6) (the identification of the coupling constants was achieved by a ^{1}H{^{31}P} decoupling experiment): δ 6.95 (t, ^{3}J(HP) = 27.1 Hz, 1H, PdCH), 6.80 (dd, *J* = 5.5, 3.2 Hz, 2H, aromatic), 6.33 (dd, *J* = 5.5, 3.2 Hz, 2H, aromatic), 3.59 (dvt, A part of an ABXX' spin system, ^{2}J(HH) = 14.9, ^{2+4}J(HP) = 3.5 Hz, 2H, PC$H^{A}H^{B}$), 2.67 (dvt, B part of an ABXX' spin system, ^{2}J(HH) = 14.9, ^{2+4}J(HP) = 6.5 Hz, 2H, PC$H^{A}H^{B}$), 1.31 (vt, ^{3+5}J(HP) = 6.8 Hz, 18H, CCH_3), 1.26 (vt, ^{3+5}J(HP) = 6.6 Hz, 18H, CCH_3). ^{13}C NMR (151 MHz, C_6D_6): δ 139.51 (vt, ^{3+4}J(CP) = 3.4 Hz, N*C*C), 119.02 (s, aromatic), 109.38 (t, ^{2}J(CP) = 1.5 Hz, PdC), 104.20 (s, aromatic), 42.07 (s, CH_2P), 35.91 (vt, ^{1+3}J(CP) = 2.1 Hz, CP), 33.95 (s, 29.84 (vt, ^{2+4}J(CP) = 4.2 Hz, CH_3), 29.59 (vt, ^{2+4}J(CP) = 3.2 Hz, CH_3). ^{31}P NMR (243 MHz, C_6D_6): δ 133.14. ESI-MS: *m/z* $[C_{25}H_{44}N_2P_2PdCl]^+$ $(M - 1)^+$ 575.17. Anal. calcd. for $C_{25}H_{45}N_2P_2ClPd$: C, 52.00; H, 7.86; N, 4.85%; found: C, 51.92; H, 7.91; N, 4.79%.

1.10. Preparation of complex 4.

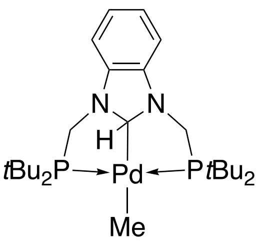

4

To a solution of **3** (0.050 g, 0.087 mmol) in benzene (10 ml) was added dropwise a solution of 1 M MeLi in Et_2O (0.0023 g, 0.104 mmol) and the mixture was stirred for 30 min. The solvent was removed under reduced pressure and the residue was extracted with diethyl ether and the yellow solution was filtered and concentrated to give a yellow solid. This solid was collected by filtration, and the solution slowly concentrated to afford yellow crystals. The air sensitive product was obtained in an overall yield of 58%. ^{1}H NMR (400 MHz, C_6D_6) (the identification of the coupling constants was achieved by a ^{1}H{^{31}P} decoupling experiment): δ 6.77 (dd, *J* = 5.4, 3.1 Hz, 2H, aromatic), 6.75 (t, *J*(HP) = 20.0 Hz, 1H, PdCH), 6.33 (dd, *J* = 5.4, 3.2 Hz, 2H, aromatic), 3.95 (dvt, A part of an ABXX' spin system, ^{2}J(HH) = 14.5, ^{2+4}J(HP) = 3.5 Hz, 2H, PC$H^{A}H^{B}$), 2.94 (dvt, B part of an ABXX' spin system, ^{2}J(HH) = 14.5, ^{2+4}J(HP) = 5.2 Hz, 2H, PC$H^{A}H^{B}$), 1.19 (vt, ^{3+5}J(HP) = 6.1 Hz, 18H, CCH_3), 1.13 (vt, ^{3+5}J(HP) = 6.3 Hz, 18H, CCH_3), 0.25 (t, ^{3}J(HP) = 5.1 Hz, 3H, $PdCH_3$). ^{13}C NMR (126 MHz, C_6D_6): δ 142.13 (br s, N*C*C), 122.47 (t, ^{2}J(CP) = 2.3 Hz, PdC), 117.62 (s, aromatic), 103.28 (s, aromatic), 47.07 (s, PCH_2), 35.63 (t, ^{1}J(CP) = 3.7 Hz, PC), 34.23 (t, ^{1}J(CP) = 2.9 Hz, PC), 29.90 (t, ^{2}J(CP) = 4.1 Hz, PC*C*), 29.73 (t, ^{2}J(CP) = 3.4 Hz, PC*C*), -15.37 (t, ^{1}J(CP) = 17.2 Hz, $PdCH_3$). ^{31}P NMR (162 MHz, C_6D_6): δ

125.26. ESI-MS: *m/z* $[C_{26}H_{47}N_2P_2Pd]^+$ $(M - 1)^+$ 555.22. Anal. calcd. for $C_{26}H_{48}N_2P_2Pd$: C, 56.06; H, 8.69; N, 5.03%; found: C, 45.62; H, 7.85; N, 3.86%. The sample was probably slightly contaminated with lithium salts and despite several attempts, we could not get better elemental analyses.

1.11. Preparation of complex 5.

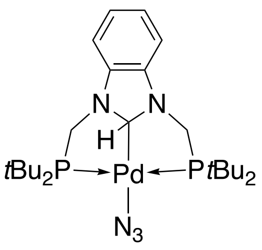

5

A mixture of **3** (0.100 g, 0.173 mmol), NaN_3 (0.079 g, 1.210 mmol) and 15-crown-5 (0.057 g, 0.260 mmol) in acetone (20 ml) was stirred overnight at room temperature, and a yellow solid precipitated. The mixture was concentrated, and the yellow solid was collected by filtration. It was redissolved in THF and excess salts were removed by filtration. The yellow solution was concentrated under reduced pressure and an air-sensitive, yellow solid was obtained, washed with toluene and diethyl ether to give pure **5** (0.085 g, yield 84%). X-ray quality crystals were obtained by slow diffusion of pentane into a toluene solution. ^{1}H NMR (400 MHz, CD_2Cl_2) (the identification of the coupling constants was achieved by a $^{1}H\{^{31}P\}$ decoupling experiment): δ 6.95 (t, $^{3}J(HP)$ = 27.5 Hz, 1H, PdCH), 6.61 (dd, *J* = 5.5 and 3.1 Hz, 2H, aromatic), 6.41 (dd, *J* = 5.5 and 3.2 Hz, 2H, aromatic), 3.93 (dvt, A part of an ABXX' spin system, $^{2}J(HH)$ = 14.9 Hz, $^{2+4}J(HP)$ = 3.7 Hz, 2H, PCH^AH^B), 3.16 (dvt, B part of an ABXX' spin system, $^{2}J(HH)$ = 14.9, $^{2+4}J(HP)$ = 6.6 Hz, 2H, PCH^AH^B), 1.49 (vt, $^{3+5}J(HP)$ = 6.6 Hz, 18H, CCH_3), 1.30 (vt, $^{3+5}J(HP)$ = 6.7 Hz, 18H, CCH_3). ^{13}C NMR (126 MHz, CD_2Cl_2): δ 138.50 (vt, $^{3+5}J(CP)$ = 1.7 Hz, NCC), 118.64 (s, aromatic), 107.38 (s, PdC, according to HSQC), 104.43 (s, aromatic), 41.85 (s, CH_2), 35.92 (vt, ^{1+3}J = 2.3 Hz, P$\underline{C}$C, this carbon does not carry a H atom according to a DEPT experiment), 33.82 (s, P$\underline{C}$C, this carbon does not carry a H atom according to a DEPT experiment), 29.65 (vt, $^{2+4}J(CP)$ = 3.5 Hz, $C\underline{C}H_3$). ^{31}P NMR (162 MHz, CD_2Cl_2): δ 137.09. ESI-MS: *m/z* $[C_{25}H_{44}N_5P_2Pd]$ $(M\text{-}1)^+$ 583.22. Anal. calcd. for $C_{25}H_{45}N_5P_2Pd$: C, 51.41; H, 7.77; N, 11.99%; found: C, 51.61; H, 7.79; N, 11.86%.

1.12. Preparation of complex 6.

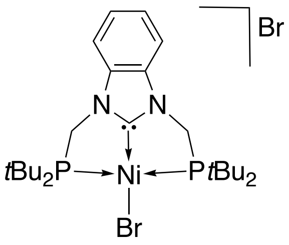

6

A mixture of **1** (0.050 g, 0.115 mmol) and [$NiBr_2$(dme)] (0.035 g, 0.115 mmol) in toluene (10 ml) was stirred overnight at room temperature. The solvent was removed under reduced pressure and the residue was dissolved in THF, then pentane was added and a light green solid precipitated. The solid was collected by filtration and pure **6** was obtained in 73.5% yield (0.055 g). X-Ray quality green crystals were grown from a saturated toluene solution. ^{1}H NMR (400 MHz, acetone-d_6): δ 7.99 (dd, J = 6.1, 3.1 Hz, 2H, aromatic), 7.51 (dd, J = 6.1, 3.1 Hz, 2H, aromatic), 5.12 (vt, ^{2+4}J(HP) = 2.4 Hz, 4H, PCH_2), 1.66 (vt, ^{3+5}J = 7.4 Hz, 36H, CH_3). ^{13}C NMR (126 MHz, acetone-d_6): δ 180.79 (t, ^{1}J(CP) = 21.4 Hz, NiC), 135.05 (vt, ^{3+5}J(CP) = 4.1 Hz, N*C*C), 125.89 (s, aromatic), 114.27 (s, aromatic), 43.73 (vt, ^{1+3}J(CP) = 13.2 Hz, PCH_2), 38.14 (vt, ^{1+3}J = 7.3 Hz, PC), 29.58 (vt, ^{2+4}J(CP) = 2.0 Hz, CH_3). ^{31}P NMR (162 MHz, acetone-d_6): δ 80.80. ESI-MS: *m/z* [$C_{25}H_{44}N_2P_2NiBr$]$^+$ (*M* - Br)$^+$ 571.1511. Anal. calcd. for $C_{25}H_{44}N_2P_2NiBr_2$: C, 45.98; H, 6.79; N, 4.29%; found: C, 43.61; H, 6.93; N, 4.16%. Despite several attempts, no better elemental analyses could be obtained.

1.13. Reaction of complex 3 with HCl in Et_2O.

A solution of 0.1 M HCl in Et_2O (0.87 ml) was added dropwise to a solution of complex **3** (0.050 g, 0.087 mmol) in benzene (10 ml) placed in an ice bath. Bubbles were formed and the solution was evaporated under reduced pressure. The solid was redissolved in CH_2Cl_2 and slow diffusion of pentane in the solution afforded white crystals of **2** (0.039 g, 80% yield).

1.14. Reaction of complex 3 with air.

A solution of complex **3** (0.050 g, 0.087 mmol) in toluene (10 ml) was exposed to the air, and its yellow colour turned purple within minutes. ^{31}P NMR spectroscopy was used to monitor the reaction by the appearance of new resonances at δ 40.16 (d, J(PP) = 420.4 Hz) and -35.32 (d, J = 420.4 Hz). After the starting material was consumed, the solvent was removed under

reduced pressure and pentane was added to extract the purple complex. This purple solution was filtered and evaporated under reduced pressure, affording the crude purple product (0.010 g). In the ESI-MS spectrum, the only peak that could be assigned was at *m/z* $[C_{25}H_{45}ClN_2P_2Pd]^+$ (**3**-H)$^+$ 576.178. This purple complex was highly soluble in organic solvents and too unstable to be slowly crystallized and it could not be further characterized.

1.15. Reaction of complex 5 with air.

The solution of complex **5** (0.050 g, 0.087 mmol) in toluene (10 ml) was exposed to the air, and its yellow colour turned purple within minutes but the reaction was slower than with **3**. The work-up was similar to that described for **3** (see above) and the crude purple complex obtained (0.011 mg) could similarly not be crystallized nor further characterized.

1.16. NMR spectra.

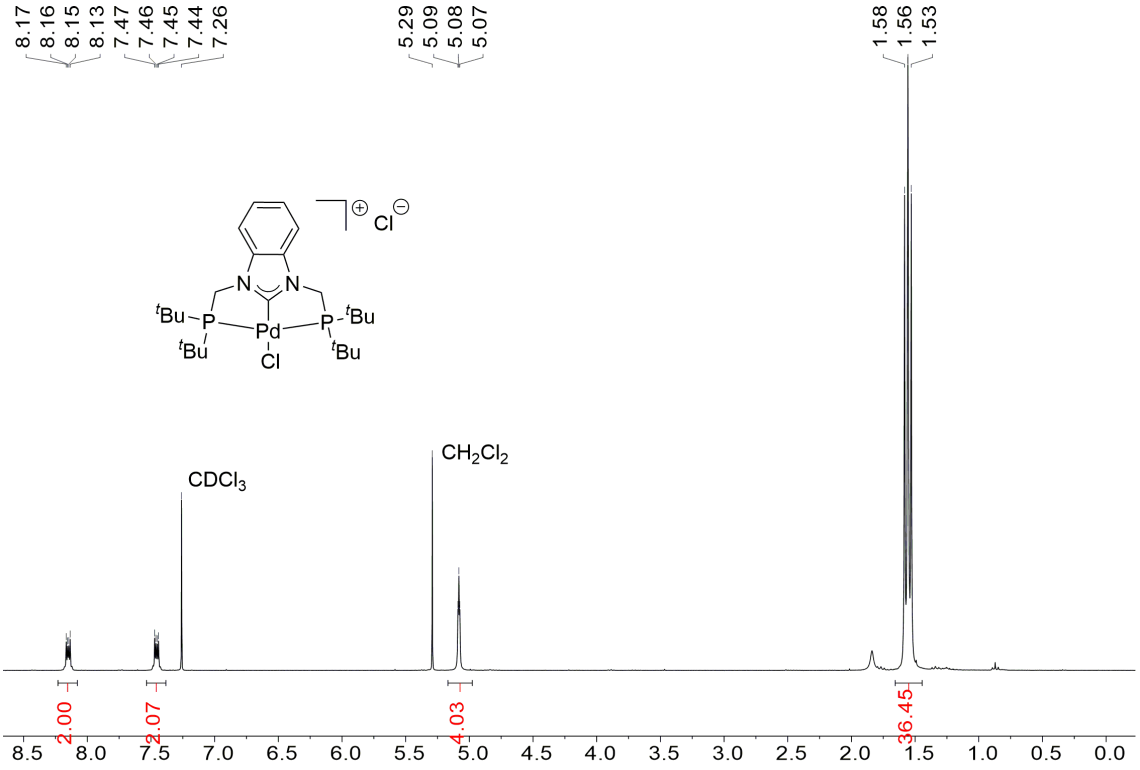

Figure S1. ^{1}H NMR spectrum of **2** in $CDCl_3$.

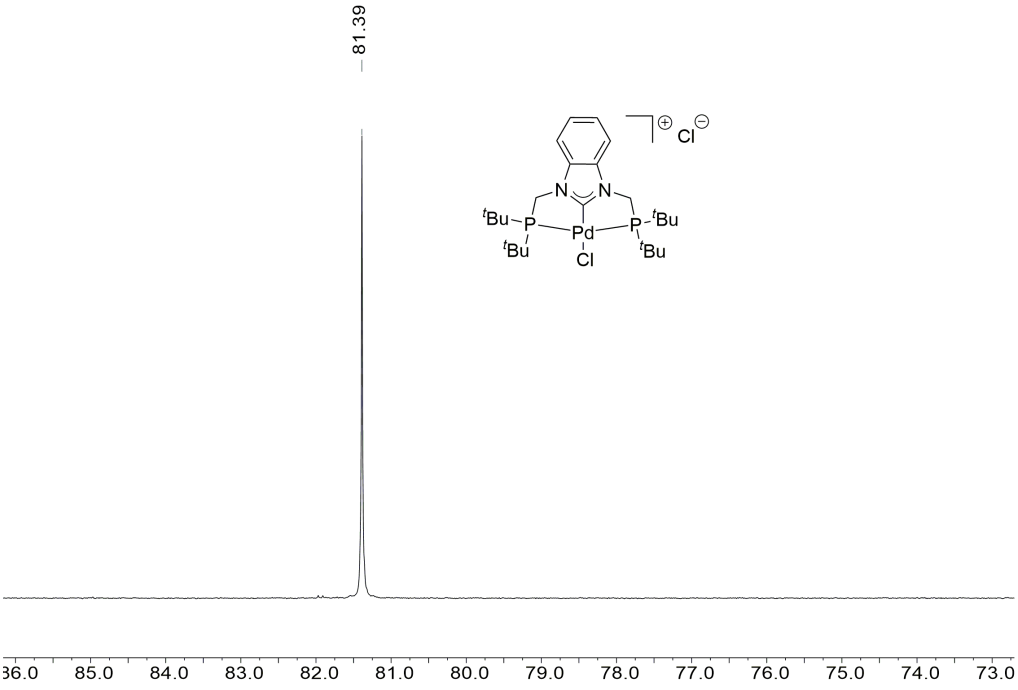

Figure S2. ^{31}P NMR spectrum of **2** in $CDCl_3$.

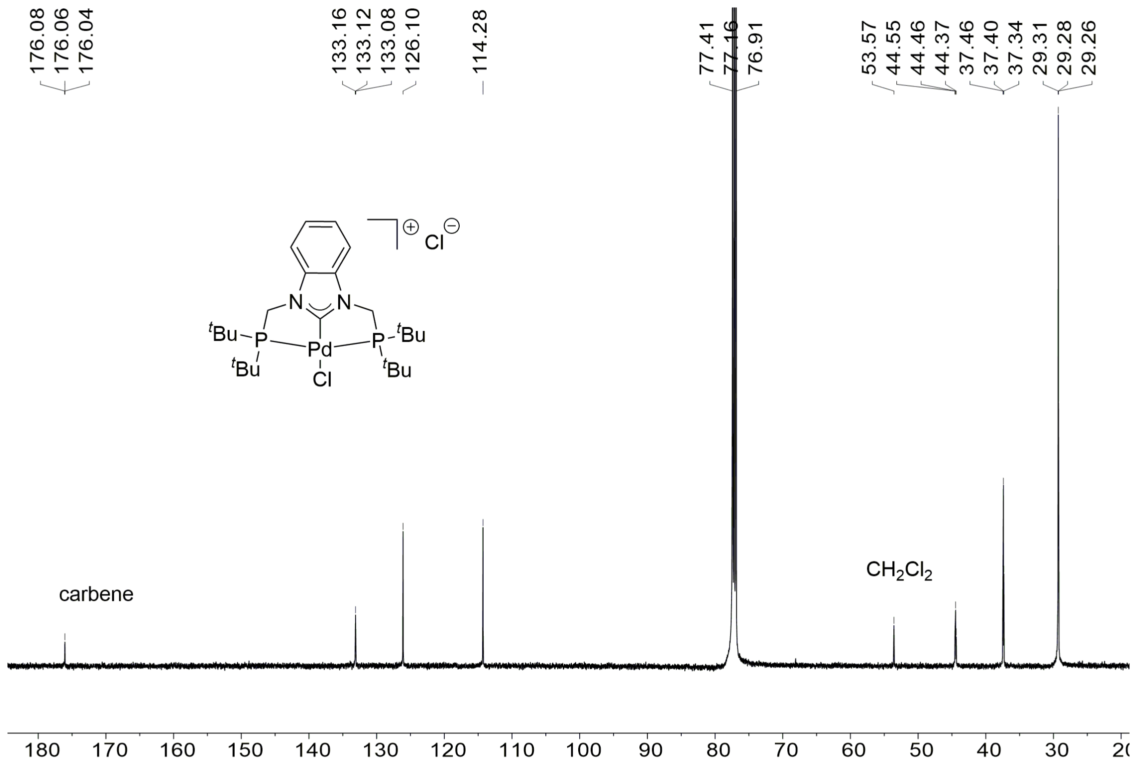

Figure S3. ^{13}C NMR spectrum of **2** in $CDCl_3$.

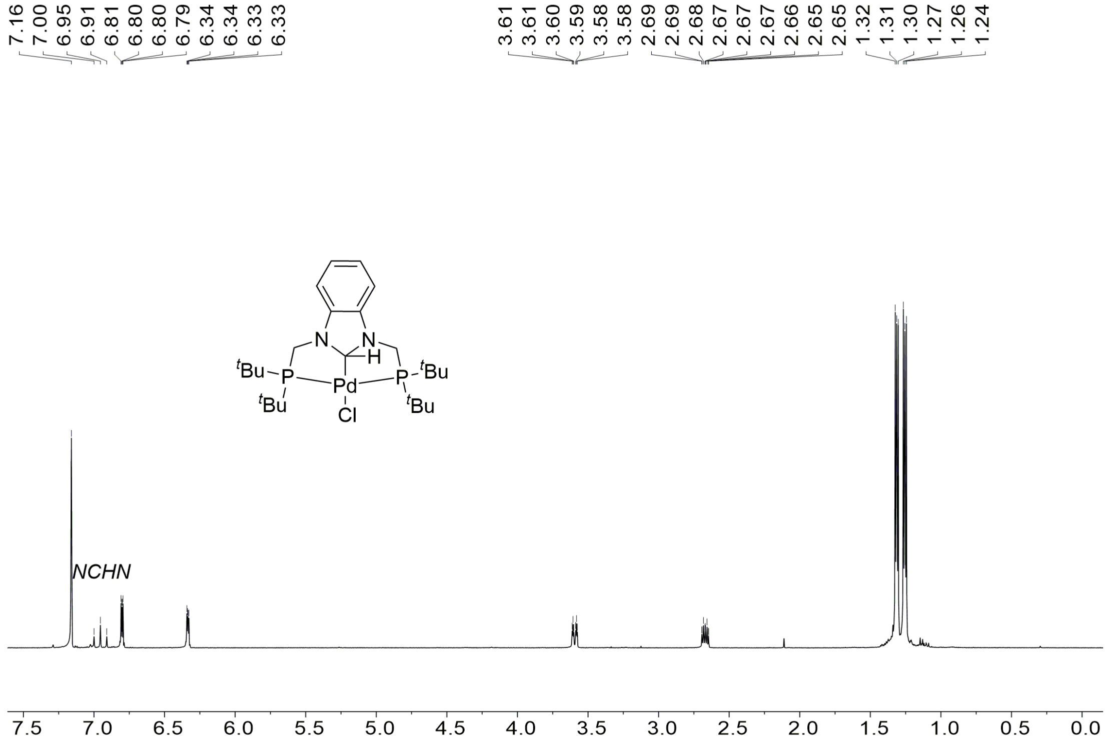

Figure S4. ^{1}H NMR spectrum of **3** in C_6D_6.

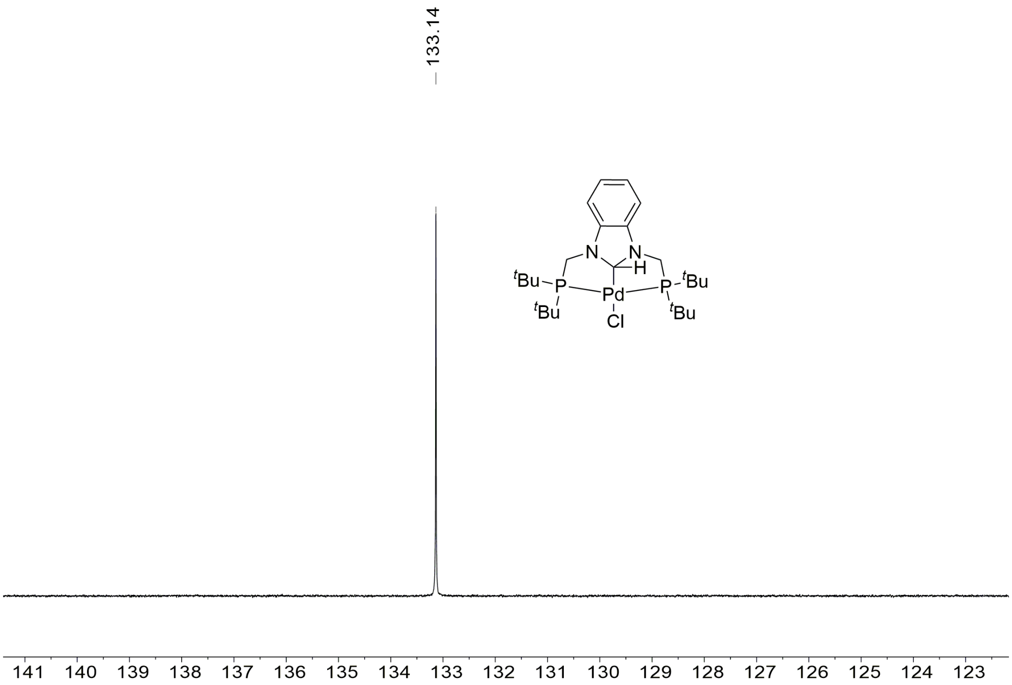

Figure S5. ^{31}P NMR spectrum of **3** in C_6D_6.

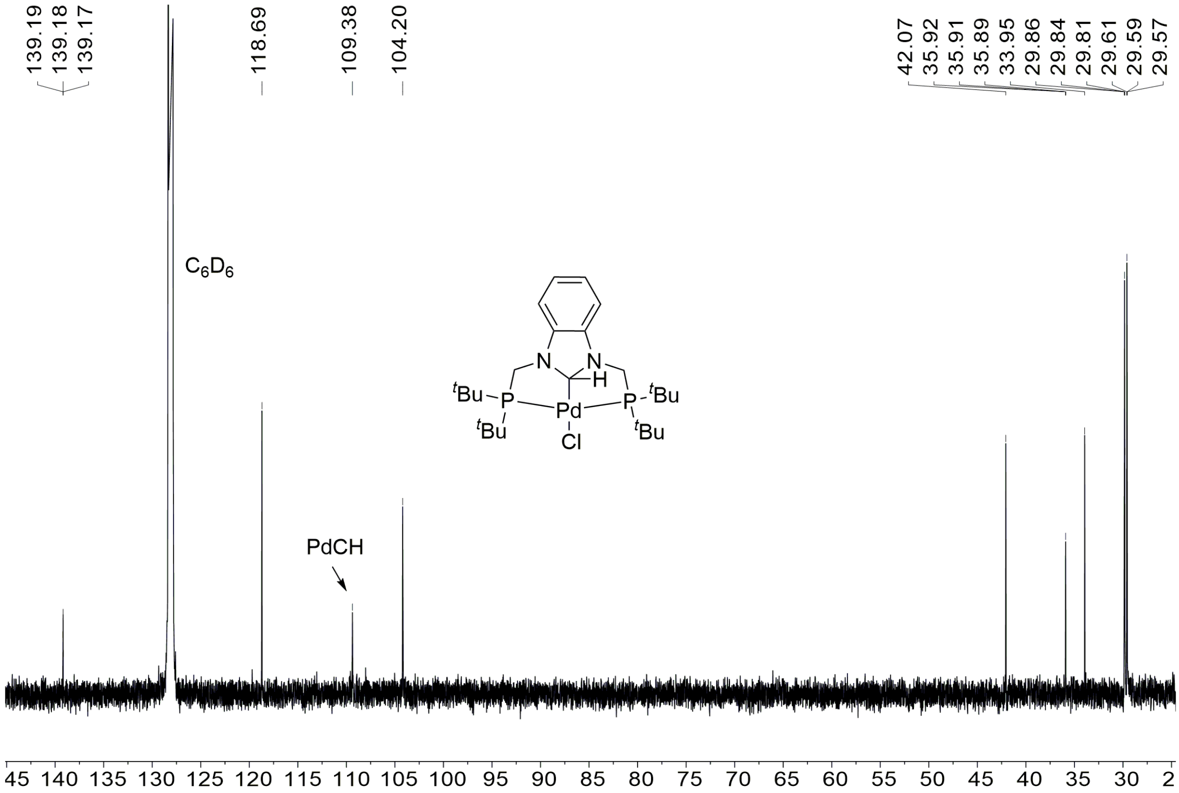

Figure S6. ^{13}C NMR spectrum of **3** in C_6D_6.

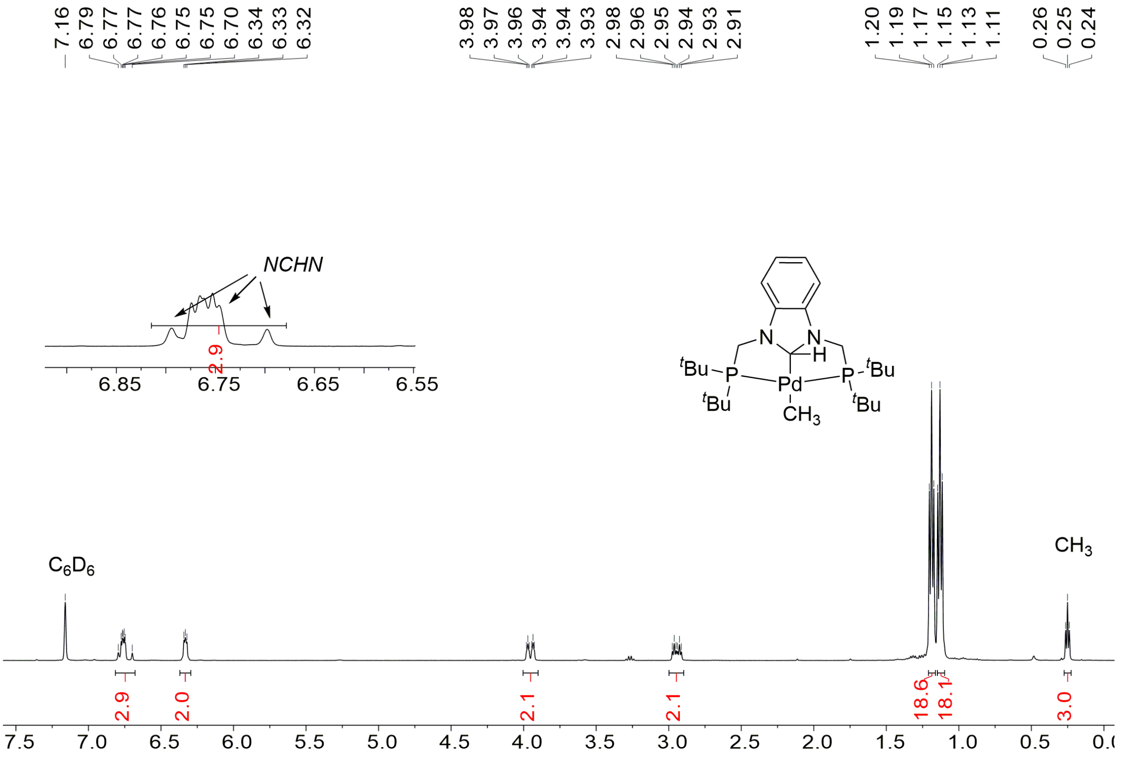

Figure S7. ^{1}H NMR spectrum of **4** in C_6D_6.

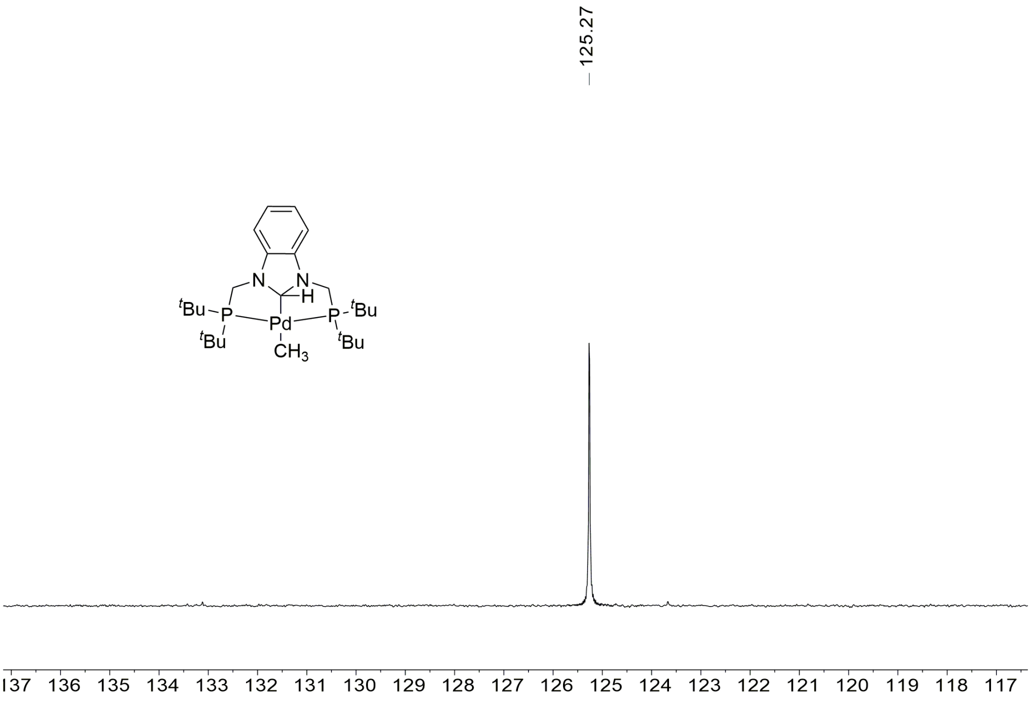

Figure S8. ^{31}P NMR spectrum of **4** in C_6D_6.

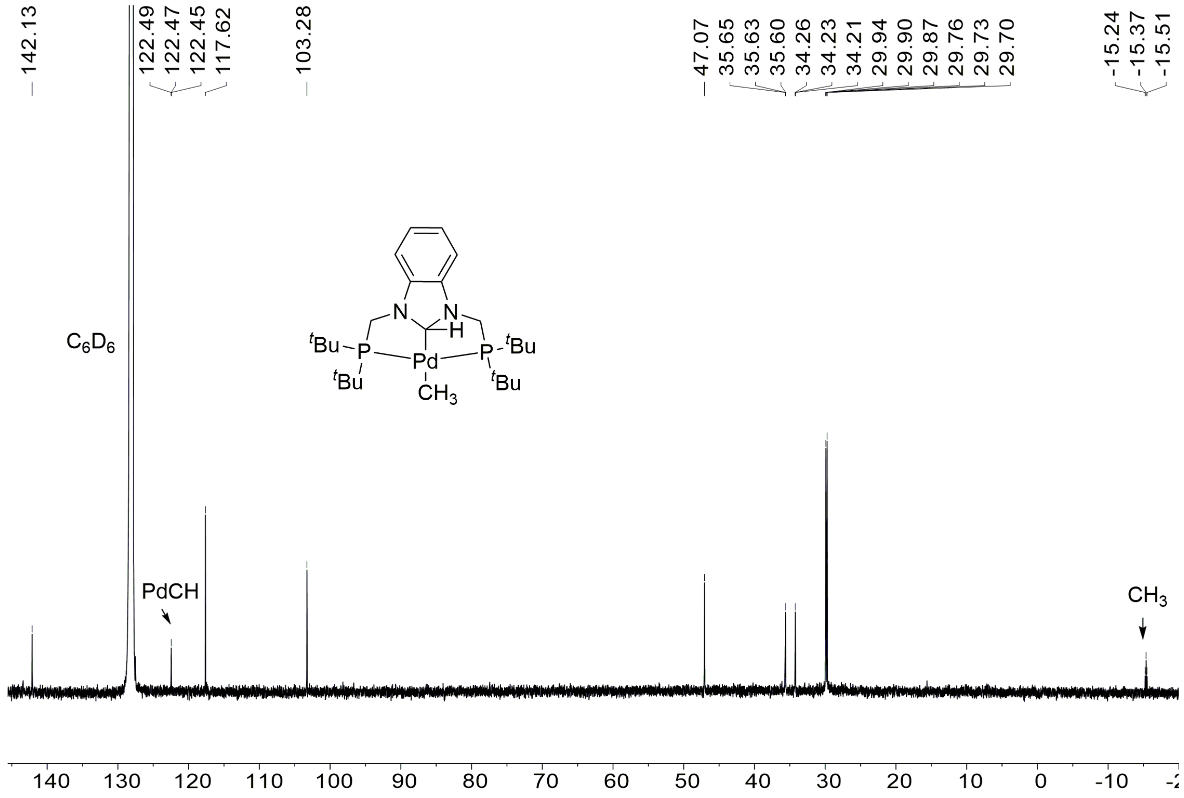

Figure S9. ^{13}C NMR spectrum of **4** in C_6D_6.

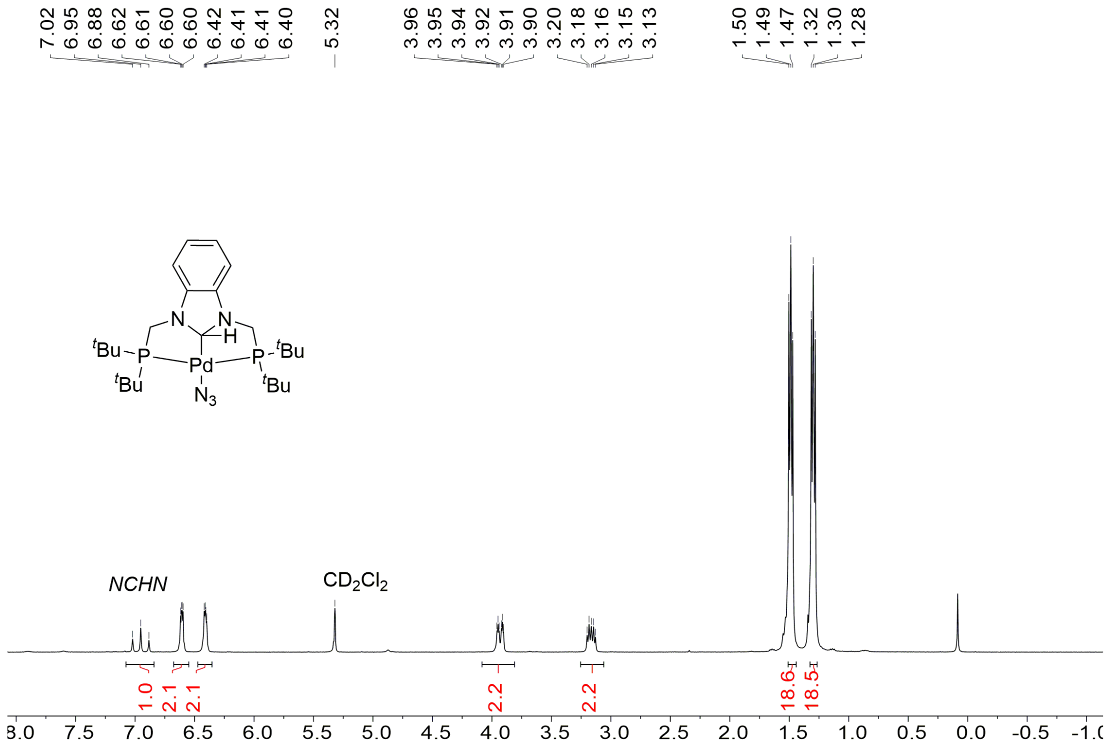

Figure S10. ^{1}H NMR spectrum of **5** in CD_2Cl_2.

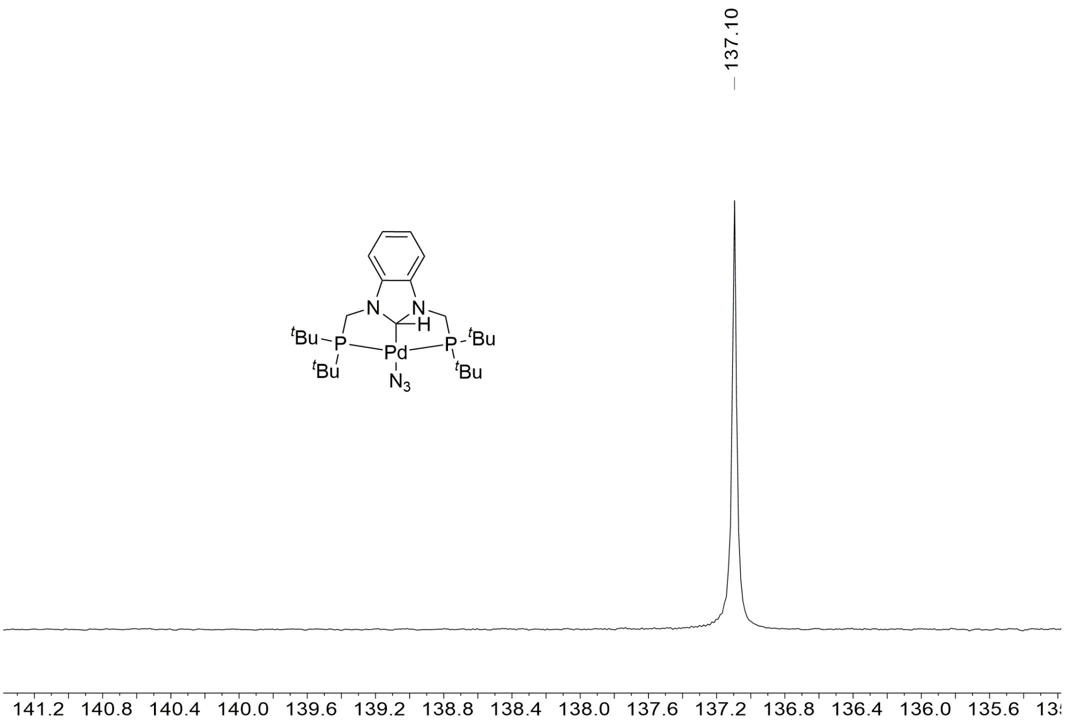

Figure S11. ^{31}P NMR spectrum of **5** in CD_2Cl_2.

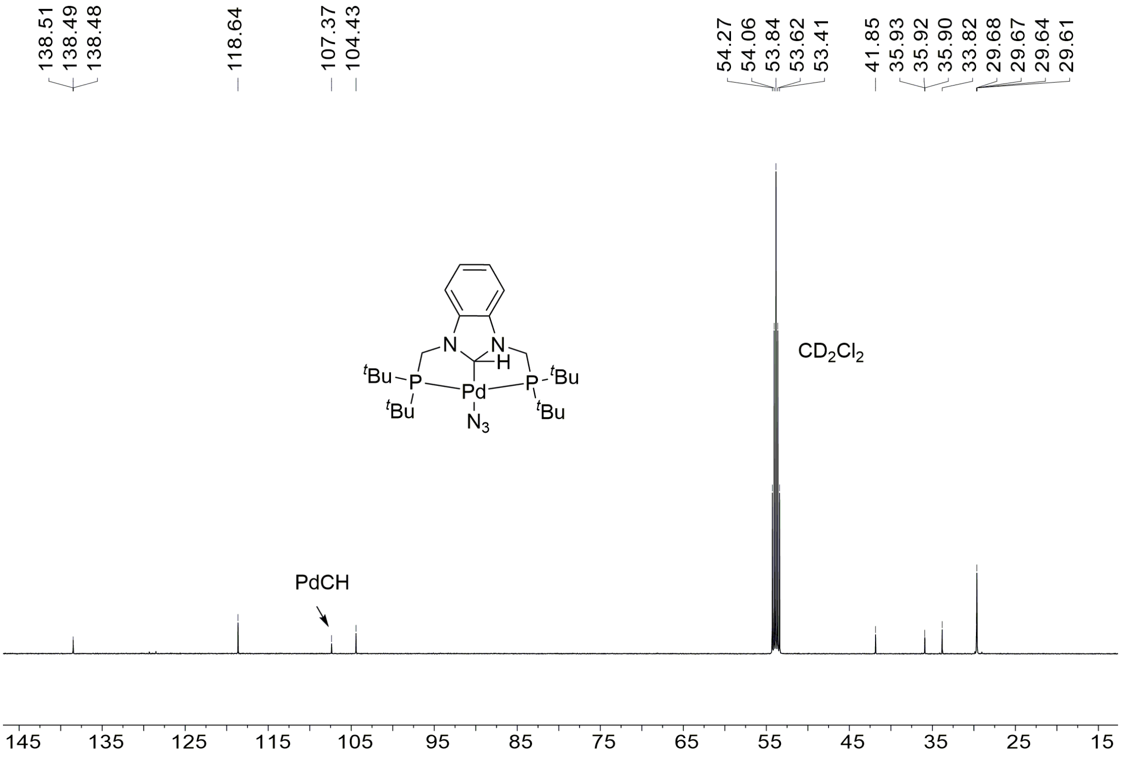

Figure S12. ^{13}C NMR spectrum of **5** in CD_2Cl_2.

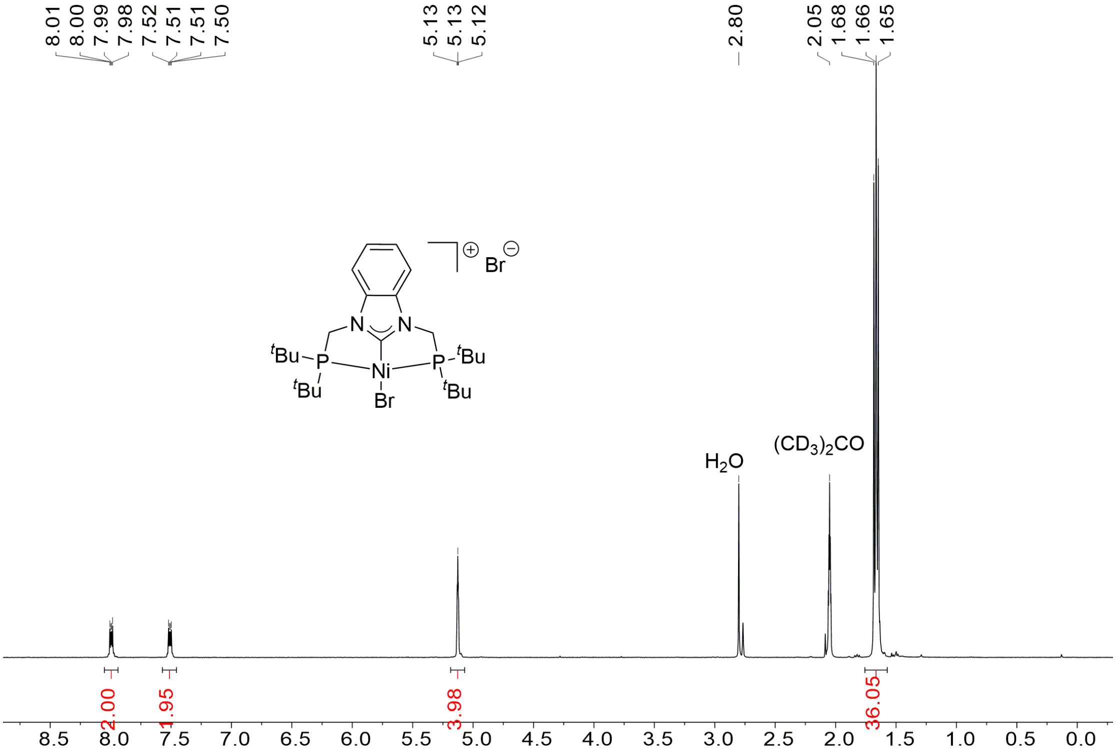

Figure S13. ^1H NMR spectrum of **6** in $(CD_3)_2CO$.

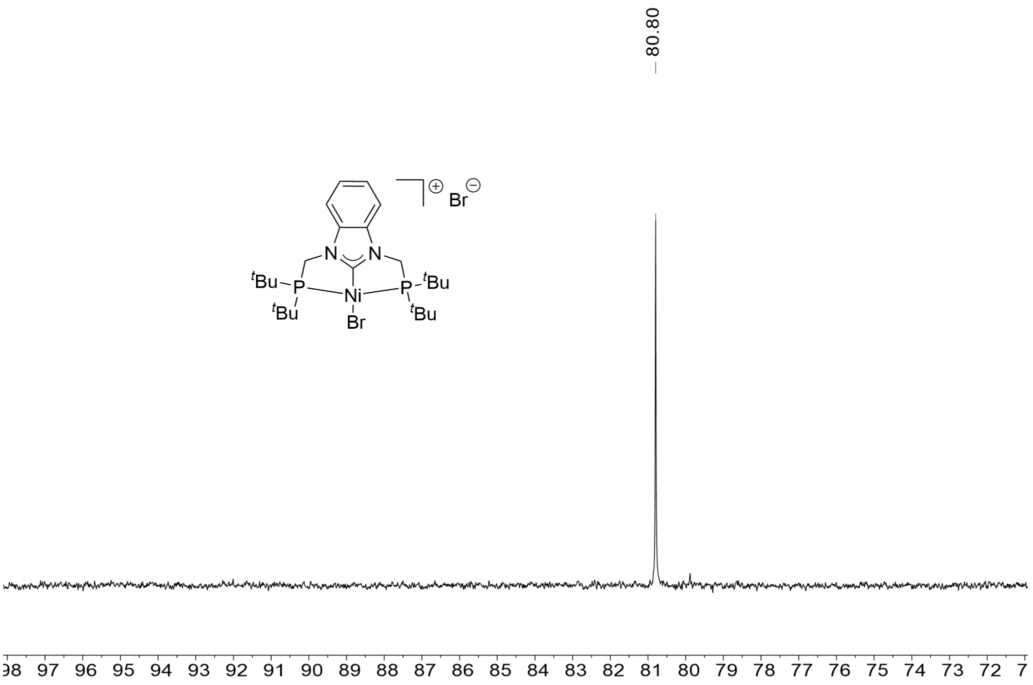

Figure S14. ^{31}P NMR spectrum of **6** in $(CD_3)_2CO$.

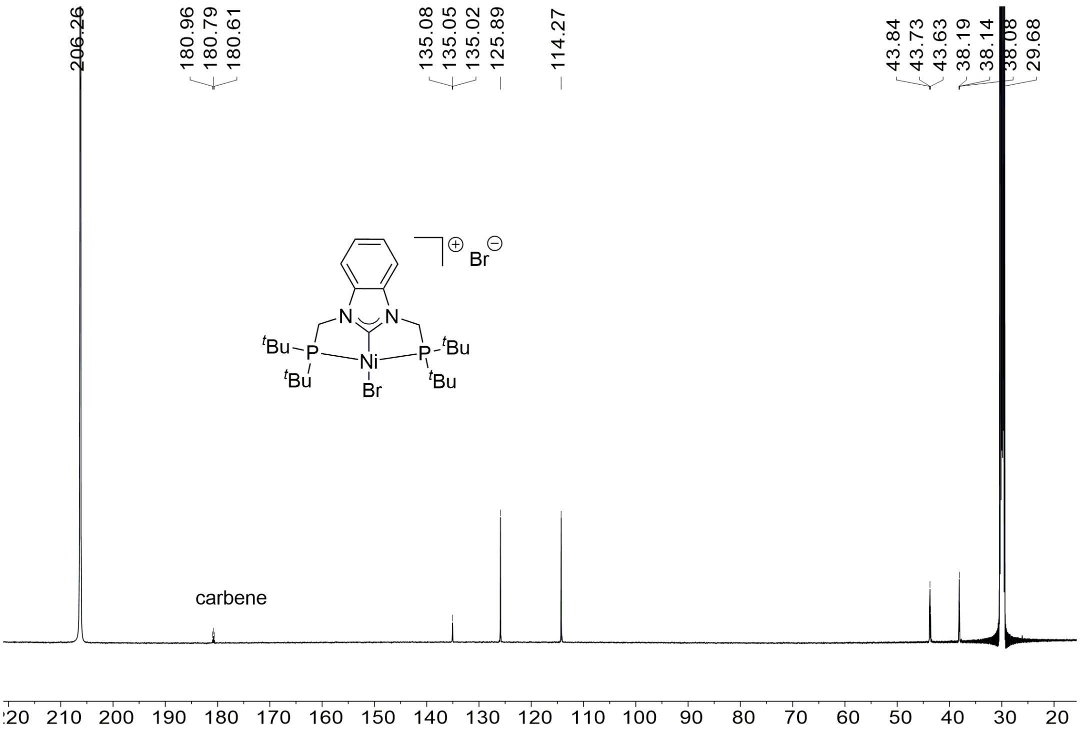

Figure S15. ^{13}C NMR spectrum of **6** in $(CD_3)_2CO$.

The methyl resonance overlaps with the solvent peaks but appears very clearly in the DEPT spectrum (see Figure S16).

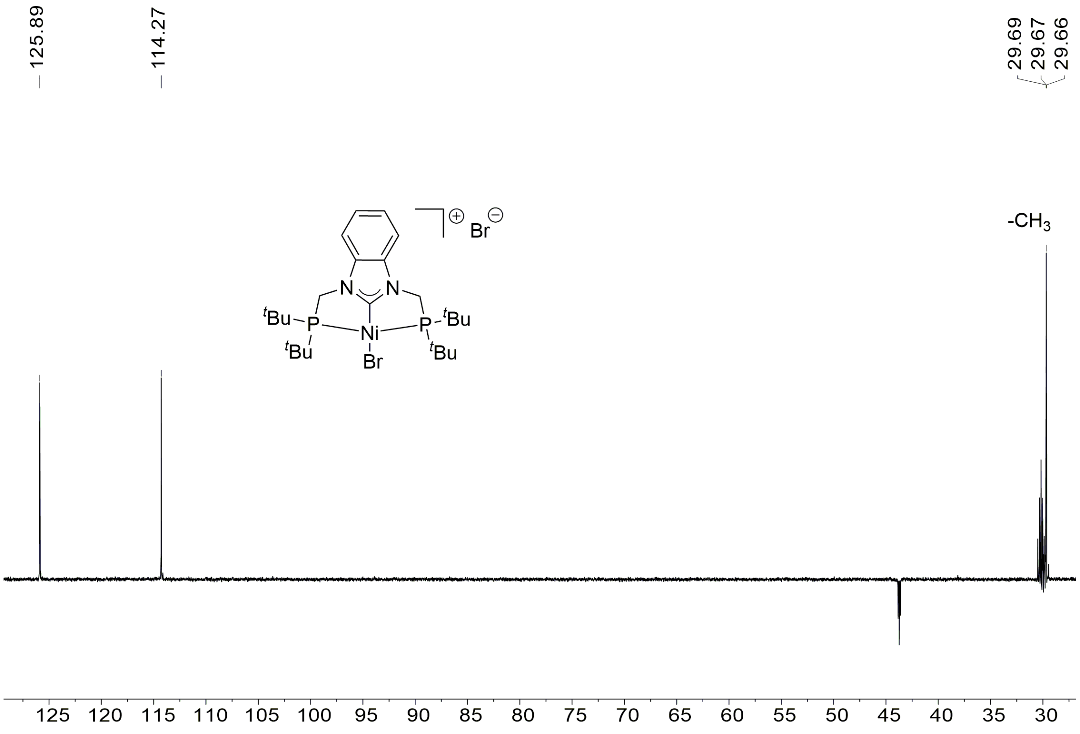

Figure S16. ^{13}C DEPT-135 NMR spectrum of **6** in $(CD_3)_2CO$.

2. X-ray crystallography

2.1. General methods.

The crystals were mounted on a glass fiber with grease, from Fomblin vacuum oil. Data sets were collected on a Bruker APEX II DUO diffractometer equipped with an Oxford Cryosystem liquid N_2 device, using Mo-Kα radiation (λ = 0.71073 Å). The crystal-detector distance was 38 mm. The cell parameters were determined (APEX2 software) from reflections taken from three sets of 12 frames, each at 10 s exposure.[4] The structures were solved by direct methods using the program SHELXS-97.[5a] The refinement and all further calculations were carried out using SHELXL-97.[5b] The H-atoms were included in calculated positions and treated as riding atoms using SHELXL default parameters. The non-H atoms were refined anisotropically, using weighted full-matrix least-squares on F^2.

2.2. Summary of crystal data.

Summary of the crystal data, data collection and refinement for the structures of **2**·CH_2Cl_2, **3**·$2C_6H_6$, and **4**, **5** and **6** are given in Tables S1and S2, respectively.

Table S1. Crystal data for compounds **1**, **2**·CH_2Cl_2, **3**·$2C_6H_6$.

	**1**	**2**·CH_2Cl_2	**3**·$2C_6H_6$
Chemical formula	$C_{25}H_2N_2P_2$	$C_{25}H_{44}Cl_2N_2P_2Pd\cdot CH_2Cl_2$	$C_{25}H_{45}N_2PdCl\cdot 2C_6H_6$
CCDC Number	2115914	2115915	2115916
Formula Mass	436.605	696.79	733.63
Crystal system	monoclinic	orthorhombic	monoclinic
a/Å	17.4667(5)	14.5088(6)	15.0262(5)
b/Å	15.5573(4)	13.2473(6)	16.9585(5)
c/Å	19.9956(5)	34.0184(15)	15.0645(4)
α/°	90	90	90
β/°	98.772(1)	90	106.6790(10)
γ/°	90	90	90
Unit cell volume/$Å^3$	5369.9(3)	6538.4(5)	3677.26(19)
Temperature/K	173.15	173.15	120.15
Space group	*C2/c*	*Pbca*	$P2_1/n$
Formula units / cell, Z	8	8	4
Absorption coefficient, μ/mm^{-1}	0.175	1.011	0.692
No. of reflections measured	51262	91552	89098
No. of independent reflections	8593	11361	11729
R_{int}	0.0288	0.0504	0.0603
Final R_1 values ($I > 2\sigma(I)$)	0.0685	0.0441	0.0390
Final $wR(F^2)$ values ($I > 2\sigma(I)$)	0.2231	0.0808	0.0785
Final R_1 values (all data)	0.0861	0.0660	0.0571
Final $wR(F^2)$ values (all data)	0.2303	0.0884	0.0880
Goodness of fit on F^2	2.354	1.136	1.075

Table S2. Crystal data for compounds **4**, **5** and **6**.

	**4**	**5**	**6**
Chemical formula	$C_{26}H_{48}N_2P_2Pd$	$C_{25}H_{45}N_5P_2Pd$	$C_{25}H_{44}Br_2N_2P_2Ni$
CCDC Number	2115917	2115918	2115919
Formula Mass	557.00	584.00	653.09
Crystal system	monoclinic	monoclinic	monoclinic
a/Å	17.9731(7)	12.3827(5)	11.4765(6)
b/Å	13.4860(5)	16.1112(6)	14.4196(9)
c/Å	11.3228(4)	14.5378(5)	22.6995(11)
α/°	90	90	90
β/°	99.3790(10)	107.476(1)	113.928(3)
γ/°	90	90	90
Unit cell volume/Å^3	2707.79(17)	2766.42(18)	3433.6(3)
Temperature/K	120.15	120.0	173(2)
Space group	$P2_1/c$	$P2_1/c$	$P2_1/c$
Formula units / cell, Z	4	4	4
Absorption coefficient, μ/mm^{-1}	0.820	0.809	2.999
No. of reflections measured	92943	81816	53850
No. of independent reflections	7310	6620	9143
R_{int}	0.0364	0.0893	0.1014
Final R_1 values ($I > 2\sigma(I)$)	0.0241	0.0349	0.0430
Final $wR(F^2)$ values ($I > 2\sigma(I)$)	0.0545	0.0731	0.0916
Final R_1 values (all data)	0.0276	0.0489	0.0984
Final $wR(F^2)$ values (all data)	0.0565	0.0807	0.1089
Goodness of fit on F^2	1.101	1.008	0.984

2.3. Crystal structure of the ligand 1.

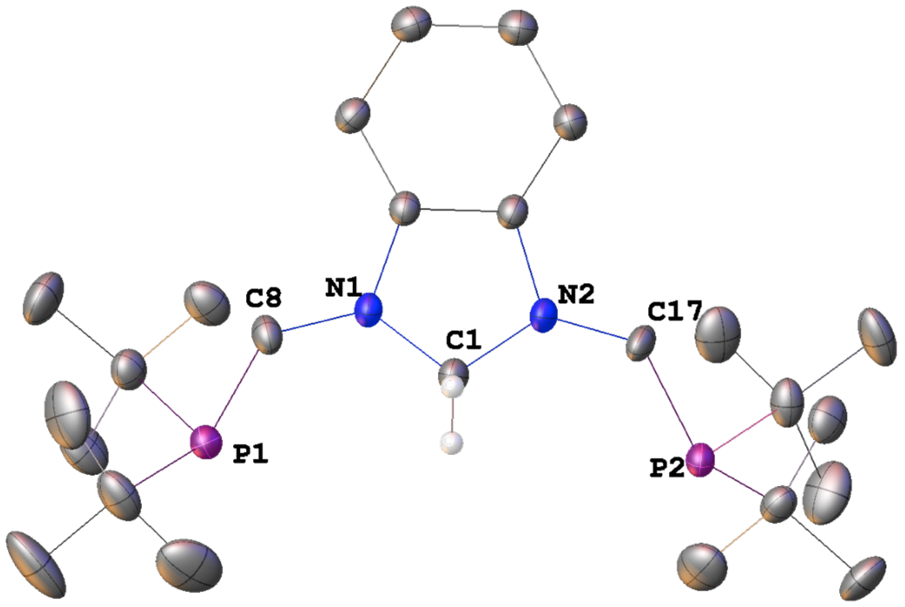

Figure S17. View of the structure of the ligand **1** with H atoms omitted for clarity, except the NCH_2N hydrogen atoms. Thermal ellipsoids at the 50% probability level. Selected bond lengths (Å) and angles (deg): C1-N1 1.4640(12), C1-N2 1.4666(13), N1-C8 1.4613(12), N2-C17 1.4616(12), C8-P1 1.8640(10), C17-P2 1.8649(10); C1-N1-C8 116.43(8), C1-N2-C17 115.92(8), N1-C1-N2 102.79(8), N1-C8-P1 111.00(7), N2-C17-P2 112.46(7).

2.4. Crystal structure of [PdCl(PC^{NHC}P)]Cl·CH_2Cl_2 (2·CH_2Cl_2).

The asymmetric unit contains one molecule of Pd complex, one chloride and one molecule of dichloromethane.

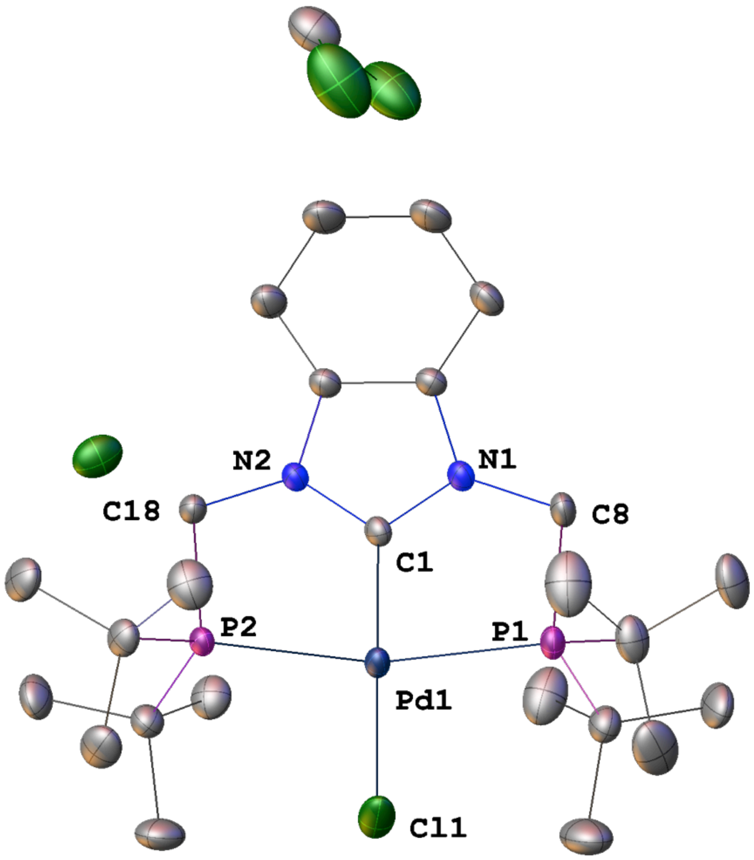

Figure S18. View of the structure of [PdCl(PC^{NHC}P)]Cl·CH_2Cl_2 (**2**·CH_2Cl_2) with H atoms omitted for clarity. Thermal ellipsoids at the 50% probability level. Selected bond lengths (Å) and angles (deg): C1-N1 1.352(3), C1-N2 1.352(3), C1-Pd1 1.940(2), Pd1-P1 2.3363(6), Pd1-P2 2.3413(6), Pd1-Cl1 2.3511(6), C8-P1 1.864(2), C18-P2 1.865(2); N1-C1-N2 107.78(19), C1-Pd1-P1 81.25(6), C1-Pd1-P2 81.54(6), C1-Pd1-Cl1 176.85(7), N1-C1-Pd1 126.36(16), N2-C1-Pd1 125.80(15), P1-Pd1-Cl1 98.78(2), P2-Pd1-Cl1 98.65(2), P1-Pd1-P2 162.22(2).

2.5. Crystal structure of $[PdCl(PC_{sp3}^{H}P)]\cdot 2C_6H_6$ ($3\cdot 2C_6H_6$).

The asymmetric unit contains one molecule of this complex and two molecules of benzene. One benzene (C26, C27, C28, C29, C30, C31) is disordered over two positions.

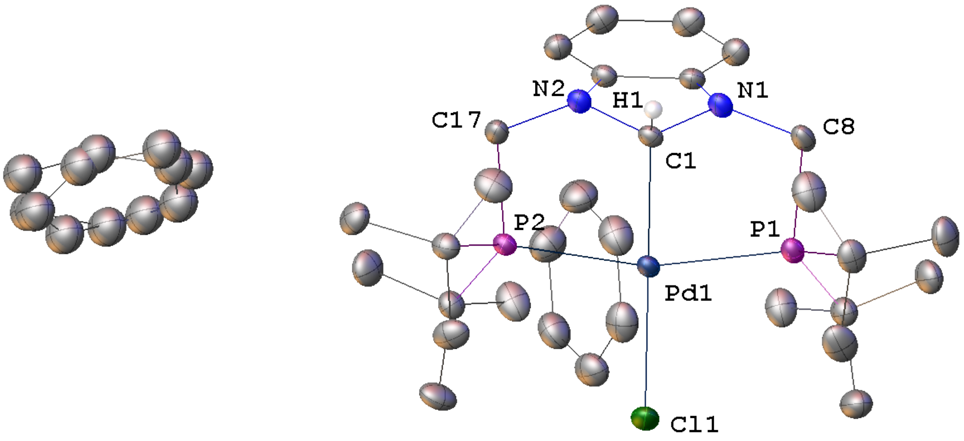

Figure S19. View of the structure of $[PdCl(PC_{sp3}^{H}P)]\cdot 2C_6H_6$ (**3**$\cdot 2C_6H_6$) with H atoms omitted for clarity except PdC1–H1. Thermal ellipsoids at the 50% probability level. Selected bond lengths (Å) and angles (deg): Pd1–C1 2.086(2), Pd1–Cl1 2.4561(6), Pd1–P1 2.3288(6), Pd1–P2 2.3219(6), C1–N1 1.448(3), C1–N2 1.452(3); C1–Pd1–Cl1 176.89(6), P1–Pd1–P2 163.34(2), C1–Pd1–P1 82.24(6), C1–Pd1–P2 82.13(6), P1–Pd1–Cl1 98.20(2), P2–Pd1–Cl1 97.72(2), N1–C1–N2 102.2(2), N1–C1–Pd1 114.58(14), N2–C1–Pd1 115.19(14).

2.6. Crystal structure of $[PdN_3(PC_{sp3}^{H}P)]$ (5)

A crystal of $C_{25}H_{45}N_5P_2Pd$, of approximate dimensions 0.090 mm x 0.100 mm x 0.100 mm, was used for the X-ray crystallographic analysis. The X-ray intensity data were measured (λ = 0.71073 Å). The integration of the data using a monoclinic unit cell yielded a total of 81816 reflections to a maximum θ angle of 27.91° (0.76 Å resolution), of which 6620 were independent (average redundancy 12.359, completeness = 99.8%, R_{int}= 8.93%, R_{sig} = 3.82%) and 5512 (83.26%) were greater than $2\sigma(F^2)$. The final cell constants $\underline{a}$ = 12.3827(5) Å, $\underline{b}$ = 16.1112(6) Å, $\underline{c}$ = 14.5378(5) Å, β = 107.4760(10)°, and V = 2766.42(18) Å^3, are based upon the refinement of the XYZ-centroids of reflections above 20 $\sigma(I)$. The calculated minimum and maximum transmission coefficients (based on crystal size) are 0.6907 and 0.7456. The structure was solved and refined using the Bruker SHELXTL Software Package, using the space group $P2_{1/c}$, with Z = 4 for the formula unit, $C_{25}H_{45}N_5P_2Pd$. The final anisotropic full-matrix least-squares refinement on F^2 with 310 variables converged at R1 = 3.49%, for the observed data and wR2 = 8.07% for all data. The goodness-of-fit was 1.008. The largest peak in the final difference electron density synthesis was 0.562 e^-/Å^3 and the largest hole was -0.805 e^-/Å^3 with an RMS deviation of 0.093 e^-/Å^3. On the basis of the final model, the calculated density was 1.402 g/cm^3 and F(000), 1224 e^-.

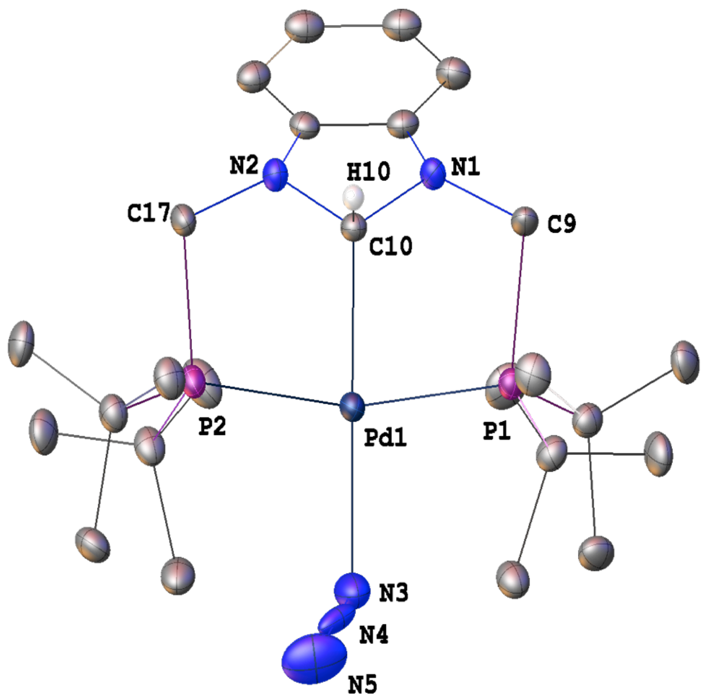

Figure S20. View of the structure of [$PdN_3(PC_{sp3}{}^{H}P)$] (**5**) with H atoms omitted for clarity except PdC10–H10. Thermal ellipsoids at the 50% probability level. Selected bond lengths (Å) and angles (deg): Pd1–C10 2.075(2), Pd1–P1 2.3070(7), Pd1–P2 2.3205(7), Pd1–N3 2.184(2), C10–N1 1.452(3), C10–N2 1.445(3); N1–C10–N2 102.38(19), N1–C10–Pd1 117.00(16), N2–C10–Pd1 115.60(16), C10–Pd1–N3 169.44(10), P1–Pd1–P2 159.87(2), C10–Pd1–P1 82.40(7), C10–Pd1–P2 82.97(7), P1–Pd1–N3 98.20(7), P2–Pd1–N3 98.72(7), Pd1–N3–N4 120.3(2), N3–N4–N5 174.2(3).

3. Computational details

All calculations were performed with GAUSSIAN 09 version D.01[6] at DFT level of theory (ωB97XD functional).[7] All atoms were described by the def2-TZVP basis set and associated pseudopotential for palladium.[8] All calculations were performed in gas phase. All structures were fully optimized and the nature of the stationary point encountered determined by frequency calculations. Minimum were characterized by a complete set of real frequencies and transition states by one and only one imaginary frequency. Gibbs free energies were extracted from this frequency calculations.

Table S3. Energies of the different structures in kcal·mol^{-1} for the double C–H bond activation relative to the separated fragments, [M(X)$_2$(L)] and ligand, L being cod for Pd and dme for Ni.

Structure	Pd	Ni
**I1**	-8.6	-10.1
**I1bis**	-10.4	-11.4
**TS1**	-9.9	-11.1
**I2**	-12.5	-11.9
**I2bis**	-16.5	-14.5
**TS2**	-3.2	-0.4
**I3**	-7.3	-3.3
**I3bis**	-19.4	-15.9
**TS3**	-10.0	3.2
**I4**	-42.7	-30.4
**I4bis**	-39.7	-29.4
**TS4**	-17.9	-17.6
**I5**	-40.9	-42.7
H_2 release	-45.5	-52.5

References

1. F. Eisenträger, A. Göthlich, I. Gruber, H. Heiss, C. A. Kiener, C. Krüger, J. Ulrich Notheis, F. Rominger, G. Scherhag, M. Schultz, B. F. Straub, M. A. O. Volland and P. Hofmann, *New J. Chem.*, **2003**, *27*, 540-550.
2. K. A. Smoll, W. Kaminsky and K. I. Goldberg, *Organometallics*, **2017**, *36*, 1213-1216.
3. A. Plikhta, A. Pothig, E. Herdtweck and B. Rieger, *Inorg Chem.*, **2015**, *54*, 9517-9528.
4. *APEX2*, *SAINT* and *SADABS*, Bruker AXS Inc.: Madison, Wisconsin, USA, **2006**.
5. (*a*) G. M. Sheldrick, *Acta Cryst.*, **1990**, *A46*, 467-473. (*b*) G. M. Sheldrick, *Acta Crystallogr., Sect. A* **2008**, *64*, 112–122.
6. Gaussian 09, Revision D.01, M. J. Frisch, G. W. Trucks, H. B. Schlegel, G. E. Scuseria, M. A. Robb, J. R. Cheeseman, G. Scalmani, V. Barone, G. A. Petersson, H. Nakatsuji, X. Li, M. Caricato, A. Marenich, J. Bloino, B. G. Janesko, R. Gomperts, B. Mennucci, H. P. Hratchian, J. V. Ortiz, A. F. Izmaylov, J. L. Sonnenberg, D. Williams-Young, F. Ding, F. Lipparini, F. Egidi, J. Goings, B. Peng, A. Petrone, T. Henderson, D. Ranasinghe, V. G. Zakrzewski, J. Gao, N. Rega, G. Zheng, W. Liang, M. Hada, M. Ehara, K. Toyota, R. Fukuda, J. Hasegawa, M. Ishida, T. Nakajima, Y. Honda, O. Kitao, H. Nakai, T. Vreven, K. Throssell, J. A. Montgomery, Jr., J. E. Peralta, F. Ogliaro, M. Bearpark, J. J. Heyd, E. Brothers, K. N. Kudin, V. N. Staroverov, T. Keith, R. Kobayashi, J. Normand, K. Raghavachari, A. Rendell, J. C. Burant, S. S. Iyengar, J. Tomasi, M. Cossi, J. M. Millam, M. Klene, C. Adamo, R. Cammi, J. W. Ochterski, R. L. Martin, K. Morokuma, O. Farkas, J. B. Foresman, and D. J. Fox, Gaussian, Inc., Wallingford CT, 2016.
7. J.-D. Chai and M. Head-Gordon, *Phys. Chem. Chem. Phys.*, **2008**, *10*, 6615-6620.
8. F. Weigend and R. Ahlrichs, *Phys. Chem. Chem. Phys.*, **2005b,** *7*, 3297-3305.
